# Xenograft and cell culture models of Sézary syndrome reveal cell of origin diversity and subclonal heterogeneity

**DOI:** 10.1038/s41375-020-01068-2

**Published:** 2020-10-26

**Authors:** Sandrine Poglio, Martina Prochazkova-Carlotti, Floriane Cherrier, Audrey Gros, Elodie Laharanne, Anne Pham-Ledard, Marie Beylot-Barry, Jean-Philippe Merlio

**Affiliations:** 1 Univ. Bordeaux, INSERM, BaRITOn, U1053, F-33000 Bordeaux, France; 2 Tumor Bank and Tumor Biology Laboratory, CHU Bordeaux, F-33000 Bordeaux, France; 3 Dermatology Department, CHU Bordeaux, F-33000 Bordeaux, France

**Keywords:** Cancer models, Cancer genetics

## Abstract

Sézary Syndrome (SS) is a rare aggressive epidermotropic cutaneous T-cell lymphoma (CTCL) defined by erythroderma, pruritis, and a circulating atypical CD4 + T-cell clonal population. The diversity of Sézary cell (SC) phenotype and genotype may reflect either plasticity or heterogeneity, which was difficult to evaluate dynamically until the achievement of long-term SC expansion. Therefore, we developed six defined culture conditions allowing for the expansion of SC defined by their phenotype and monoclonality in four of seven SS cases. Engraftment of SC through the intrafemoral route into immunodeficient NOD.Cg-Prkdc(scid)Il2rg(*tm1Wjll*)/SzJ (NSG) mice was achieved in 2 of 14 SS cases. Secondary xenograft by percutaneous injection mimicked most of the features of SS with dermal infiltration, epidermotropism, and blood spreading. These models also allowed assessing the intra-individual heterogeneity of patient SC. Subclones sharing the same TCR gene rearrangement evolved independently according to culture conditions and/or after xenografting. This clonal selection was associated with some immunophenotypic plasticity and limited genomic evolution both in vitro and in vivo. The long-term amplification of SC allowed us to develop eight new SC lines derived from four different patients. These lines represent the cell of origin diversity of SC and provide new tools to evaluate their functional hallmarks and response to therapy.

## Introduction

Sézary Syndrome (SS) is a rare and aggressive cutaneous T-cell lymphoma (CTCL) defined by erythroderma with a circulating atypical CD4 + T-cell clone. By definition, blood involvement is high with B2 stage (i.e., CD4 + CD7- cells or CD4 + CD26- cells or other aberrant phenotype ≥1000/μL in the presence of a relevant T-cell clone in blood) [1]. New criteria to assess the B2 stage have been evaluated to achieve interlaboratory reproducibility for SS diagnosis [2]. The ability of Sézary cells (SCs) to circulate is supported by their central memory T-cell phenotype (CCR7 + CD45RO + CD45RA-CD62L+) and their skin homing properties based on the expression of specific molecules, such as CCR4 and CLA [3]. However, recent data suggest that SCs are not restricted to a central memory phenotype but are either heterogeneous in their cell of origin or differentiation stage according to the expression of markers characteristic of stem cell memory (T_SCM_), central memory (T_CM_), effector memory (T_EM_), and naive or transitional memory (TTM) T cells [4, 5]. Their heterogeneity at the single-cell level is also important to understand variable responses to treatments, such as HDAC inhibitors [6]. In addition to their phenotypic diversity, SCs show a heterogeneity of mutations involving oncogenes, tumor suppressor genes, or epigenetic modulators with a mixture of drivers and passenger events at various rates [7–9]. Their different allelic frequencies would suggest that the monoclonal T-cell population may contain different subclones with specific properties according to quiescence, stemness, or proliferation, as supported by a heterogeneous single-cell transcriptional profile of CTCL [10].

So far, the biological characterization of SCs has been impaired by the lack of reproducible in vivo and in vitro models. A murine model was developed after injection of MBL2 T lymphoma cells followed by inflammatory peptide inoculation into the ears, resulting in a local development of lymphoma [11]. More recently, an original murine model of IL-15 transgenic mice harboring some CTCL features within 4 to 6 weeks of birth (including full-body, scaly erythematous plaques/patches, exfoliative dermatitis, ulcerations, severe pruritus, and increases in peripheral white blood cell) was developed [12]. For human models, few HTLV-1 negative SC lines are available (Sez/SZ4, HUT78/H9, and SeAx) and do not represent SC diversity [13–15]. Moreover, a fully relevant model of SS with both skin and blood involvement was never achieved using these cell lines, despite the use of different sites of injection and mouse recipients (NOD SCID IL2γc-/-, CB17 SCID beige) [16–18]. Only two groups recently obtained patient-derived xenograft (PDX) from three SS cases with migration to the skin and epidermotropism after intravenous injection of SC [19, 20]. Among established cell lines, percutaneous injection of Sez4 and SeAx cell lines failed to engraft, whereas HUT78 cell injection led to local tumorigenesis without cell migration to other tissues [21]. Recently, we developed a robust model of intrahepatic injection of several CTCL lines achieving both successful hepatic engraftment for 80% of cell lines and dissemination to several organs such as spleen and kidneys, but without skin infiltration [22].

In this study, we established new in vitro culture conditions and a PDX model to achieve SC expansion from fresh patient cells. These models confirmed genomic and immunophenotypic interindividual diversity and revealed different subpopulations among original clonal T cells within the same patient. Establishing eight new SC lines corresponding to several SC differentiation stages also provides novel tools to characterize SC hallmarks.

## Materials and methods

### Patient characteristics and human Sézary sample processing

Blood samples from 14 adult patients with SS T4NxMxB2 stage (at one time of illness) were collected at the Oncodermatology Department (Bordeaux, France) (Supplementary Table S1). Patients provided informed consent in accordance with the Declaration of Helsinki and national ethics rules. The institutional review board of our institution approved the manipulations of Sézary samples (DC-2008–412). Peripheral blood mononuclear cells (PBMCs) were isolated by Pancoll centrifugation, immuno-phenotyped, and used directly or frozen in fetal calf serum containing 10% DMSO.

### Flow cytometry

Antibodies used for immunolabeling are described in the Supplementary methods. SCs were identified based on TCRVβ + CD4 + CD3 + CD8- cell surface expression in normal mature T cells by Fluorescence-Activated Cell Sorting (FACS) using a Canto II cytometer (BD Biosciences) with FACS Diva Software. For in vitro manipulation, SCs were sorted using an ARIA II cell sorter (BD Biosciences, Le Pont de Claix, France).

### TCRγ rearrangements

The human TCR gamma gene rearrangement was studied using the BIOMED-2 protocol, as described previously [23].

### Mouse models

PBMCs from primary patient samples were injected intrafemorally or percutaneously (1 × 10^6^ cells/mouse unless otherwise specified) in immunodeficient NOD.Cg-Prkdc(scid)Il2rg(tm1Wjll)/SzJ (NSG) mice (from the Jackson Laboratory). The monitoring of mice engraftment and tissue analyses are described in the Supplementary materials and methods.

### Culture of patient SCs

SCs purified by FACS were co-cultured with or without MS5 or MS5-Delta Like 1 (MS5-DL1) stromal cells, as described previously [24]. MS5 and MS5-DL1 cells were kindly provided by Dr F Pflumio (U1274 INSERM, Fontenay-aux-Roses, France). These culture conditions were adapted by adding cytokines (mix A: IL-2 [25 ng/mL], IL-4 [17.5 ng/mL], phytohemagglutinin (PHA) 1%, and mix B: IL-7 [10 ng/mL], IL-15 [10 ng/mL]). Cells were collected weekly from individual wells, counted, and then re-plated in the appropriate culture conditions for 4 weeks. To obtain SC lines, we selected the culture condition providing the highest proliferation rate for further expansion for at least 6 weeks.

### SC line treatments

One million cells from SC lines (L1, L2, L4, L5, and L7) were plated per six-plate well and treated for 48 h with romidepsin (10 nM), doxorubicin (20 nM), and vorinostat (3 µM) [25, 26]. For Annexin V+/Hoechst- (A+/H−), Annexin V-/Hoechst + (A−/H+), and Annexin V+/Hoechst+ (A+/H+) cell detection, cells were stained with Annexin V-PE (BD Biosciences) according to the manufacturer’s recommendations and Hoechst 33342 was added 5 min before sample acquisition. FACS as mentioned above was used to evaluate A+/H-, A-/H+, or A+/H+ cells, which are indicators of early apoptosis, necrosis and late apoptosis, respectively.

### Immunohistochemistry

Immunohistochemistry and Hematoxylin–Eosin–Saffron staining were performed as described previously [22] and in the Supplementary materials and methods.

### Multicolor fluorescence in situ hybridization (mFISH) karyotyping

Cultured cells were harvested using the cytogenetic standard protocol for metaphase lymphocytes and mFISH experiments were performed as described previously [27–29].

### Oligonucleotide array-based CGH

Oligonucleotide array-based CGH was conducted as described previously [30].

### Lymphopanel analyses

Lymphopanel sequencing was performed on seven patients and eight SC lines using the IonS5 (ThermoFisher Scientific). This lymphopanel was designed with Ion Ampliseq technology (ThermoFisher Scientific, Life Technologies, Les Ullis France) to identify mutations within frequently altered genes in SS and peripheral T-cell lymphoma (*ARID1A*, *CARD11*, *CCR4*, *CD28*, *DNMT3A*, *FAS*, *FASN*, *IDH2*, *JAK3*, *KDM6B*, *MLL3/KMT2C*, *PLCG1*, *RHOA*, *SETD1B*, *STAT3*, *STAT5B*, *TET2*, *TP53*, *ZEB1*) [8, 9, 31]. Data processing and protocols are described in the Supplementary materials and methods.

### Statistical analyses

Statistical significance of compared measurements was determined using the Mann–Whitney nonparametric test using GraphPad Prism (GraphPad Software, Inc. La Jolla, CA, USA).

*P* values < 0.05 were considered significant.

## Results

### Patient SC growth in defined culture conditions

**Fig. 1:**
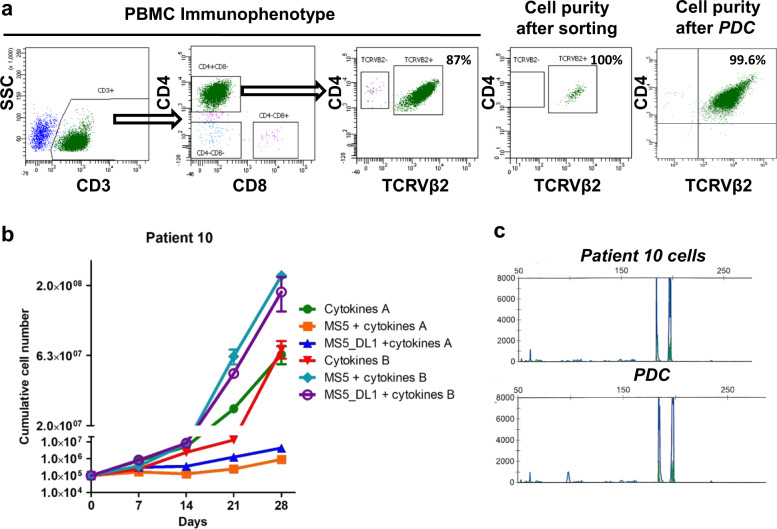
Patient Sézary cells (SCs) in vitro expansion in defined media. **a** Characterization of patient #10 SCs according to TCRVβ2, CD3, CD4, and CD8 expression. Dot plots representing the immunophenotype of PBMC, the purity of tumor TCRVβ2 + CD3 + CD4 + CD8-cells after flow cytometry cell sorting and the identification of SC purity after culture (patient-derived culture, PDC) by detection of TCRVβ2 + CD4+ cells using FACS. **b** Primary cultures of patient #10 SCs for 28 days after SC sorting. Cells were cultured on six different culture conditions and counted every week. **c** Identity of TCRγ gene rearrangement between original SCs of patient #10 and PDC was determined by the Biomed-2 protocol and capillary fragment analysis.

In seven patients, SCs were identified by their CD3 + CD4 + CD8- phenotype and the monotypic expression of a TCRVβ variant. The proportion of tumor cells was already dominant in unsorted blood samples (88 ± 6%) and purity of the tumor population reached 99 ± 2% after sorting in all samples (Fig. 1a, Supplementary Table S2). SCs were sorted based on TCRVβ2 + CD3 + CD4 + CD8- phenotype and then plated with or without stromal MS5/MS5-DL1 cells [24]. Medium was implemented with or without cytokines known to activate T cells [32–35]. Among the six culture conditions tested, four of seven patient samples were amplified in vitro in at least one culture condition that was patient-specific (Supplementary Table S2). For two patients (#2 and #5), SCs plated without stromal cells showed the highest expansion compared to other conditions (Supplementary Fig. S1A, B). The SCs of patients #6 and #10 were amplified more rapidly when plated on stromal cells than without them (Fig. 1b, Supplementary Fig. S1A). Expression of a common TCRVβ variant and presence of the same monoclonal TCRγ rearrangement of patient-derived cultured (PDC) cells supported that expanded SCs originated from patient SCs (Fig. 1a, c, Supplementary Fig. S1C). The original SC phenotype was not predictive of cell growth within a defined culture condition, but all amplified samples expressed the TCRVβ2 variant (Supplementary Table S2). Altogether, using six parallel culture conditions was required to achieve at least one amplification of SCs in 57% of patients tested (four of seven cases).

### In vivo modeling of SS

**Fig. 2:**
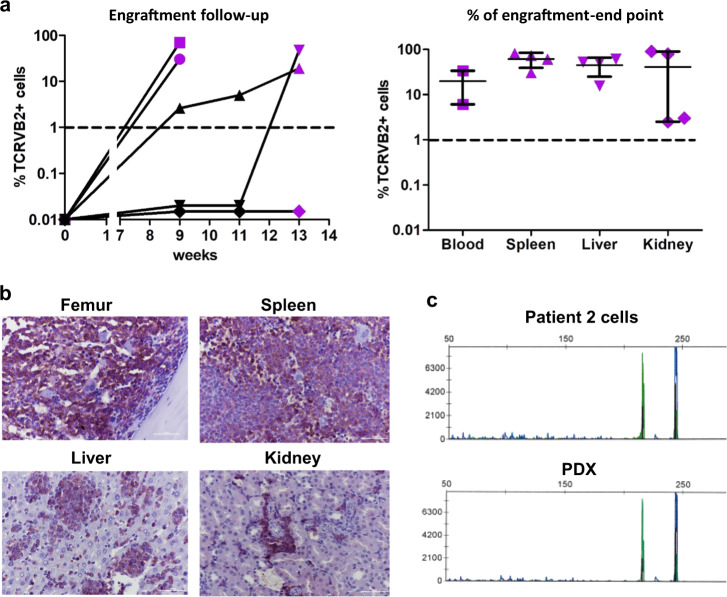
Patient SCs expansion in an immunodeficient mice model. **a** 1 × 10^6^ patient # 2 SCs (TCRVβ2 + CD3 + CD4 + CD8−) were injected in NOD SCID IL2Rγc−/− (NSG) immunodeficient mice and percentage of tumor cells was determined in bone marrow (BM) by FACS from week-9 and then every 2 weeks. Mice were sacrificed when percentage of SC in BM reached at least 50% or when mice shown signs of illness. Purple dots represent the percentage of engraftment in femur at sacrifice. For each tissue median and range are shown. **b** Identification of human cells (HLA-ABC) by immunohistochemistry in femur, spleen, liver, and kidney. **c** Identity of TCRγ gene rearrangement between original SCs and PDX cells was determined by the Biomed-2 protocol and capillary fragment analysis.

To improve the in vivo models of SS using the few available SC lines [16–18], we established a new standardized model using patient cells. PBMCs from 14 patient samples (Supplementary Table S1) were injected intrafemorally into NSG mice (*n* = 5 minimum) and the percentage of TCRVβ characteristic of tumor cells was analyzed from bone marrow (BM) aspiration. Only 14.3% (patients #2 and #10 out of 14 cases) of Sézary samples engrafted, even after 6 months of monitoring (Supplementary Table S3). For patient #2, four of five mice developed the disease from week 9 to 13 with different speeds of engraftment (Fig. 2a), whereas patient #10 cells engrafted after 18 weeks (Supplementary Fig. S2A, B). At sacrifice, patient SCs invaded different tissues such as the opposite femur, spleen, liver, kidneys, and blood, but without any skin involvement (Fig. 2a, b, Supplementary Figs. S2A, S3). We also confirmed that SCs were derived from original patients’ cells (Fig. 2c, Supplementary Fig. S2B). SCs were able to reinitiate the disease in secondary xenografts and to engraft more rapidly than the primary xenograft, at 5 vs. 9 weeks, respectively (Supplementary Fig. S4A). The infiltration of different tissues by SCs appeared homogeneous in cell percentage (Supplementary Fig. S4B) and was confirmed by immunostaining (Supplementary Fig. S4C) and clonality assessment (data not shown). The ability of SCs to reinitiate the disease in secondary recipients also supported their lymphoma-initiating property.

**Fig. 3:**
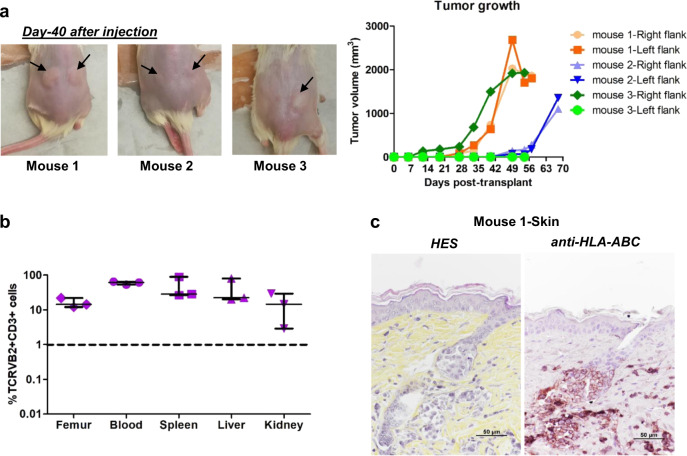
Renewal of patient SCs in secondary transplant at skin site. 0.4–2 × 10^6^ tumor cells (TCRVβ2 + CD3 + CD4 + CD8-) from patient #2 derived from primary xenograft were injected percutaneously in secondary NSG mice and percutaneous tumor volume was measured during 8–10 weeks after transplantation. **a** Pictures showing cutaneous tumors in mice. Arrows indicate the site of tumor formation. Tumor size were measured weekly until the sacrifice. The graph represents the evolution of tumor volume. **b** Mice were sacrificed when the skin tumor volume reached 2000 mm^3^. The cells from different tissues were prepared and percentage of TCRVβ2 + CD3 + cells analyzed by FACS. Graph represents the percentage of TCRVβ2 + CD3 + cells in femur, blood, spleen, liver and kidney. For each tissue, median and range are shown. **c** Skin lesion of mouse 1 was analyzed by HES staining and human cells identified by HLA-ABC immunostaining in skin section. A marked dermal infiltration and regular pilotropism of HLA-ABC positive cells was observed in all mice.

To obtain skin lesions, we also performed secondary xenografts of patient #2-derived cells through the percutaneous route, which is generally used to monitor local tumorigenesis as the long-established SS cell lines do not spread out into the bloodstream [18]. Local tumorigenesis was achieved in 100% of animals (Fig. 3a) and involved spreading to different tissues in all mice (Fig. 3b). Interestingly, the percentage of tumor cells in the blood reached around 60% in all animals (Fig. 3b, Supplementary Fig. S4D), which contrasts with its absence after HUT78 percutaneous engraftment (Supplementary Fig. S5). While skin lesions remained localized around the injection site, histopathological analyses showed that infiltration of the dermis was associated with regular pilotropism and epithelial migration of some SCs (Fig. 3c). Such preclinical model may represent a valuable tool to analyze both epidermotropism and blood spreading mechanisms.

### Establishment of SC lines from PDC and PDX

**Fig. 4:**
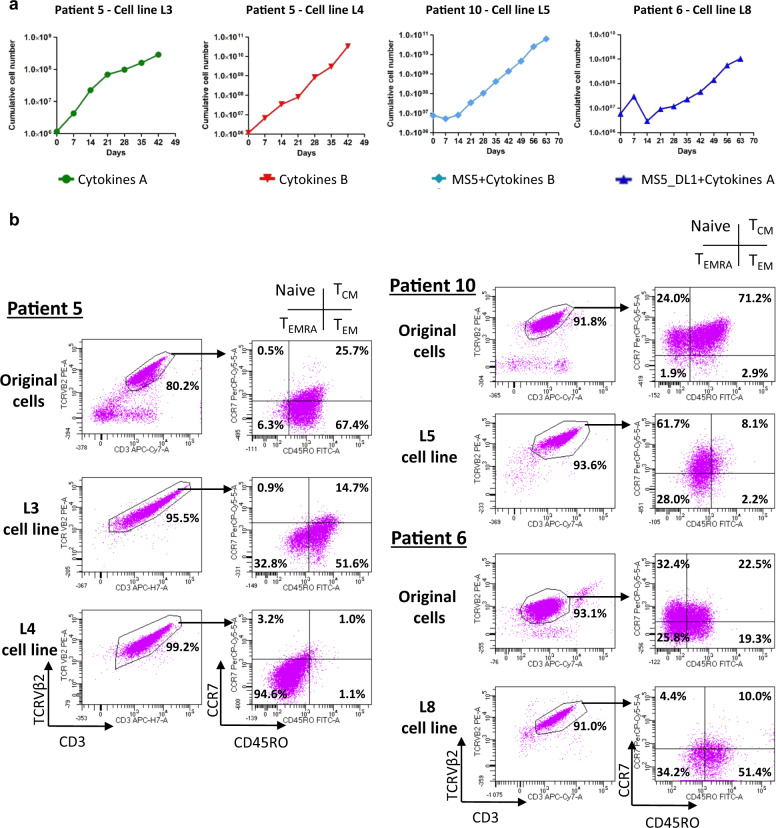
Establishment of new SC lines from cultures. **a** After primary cultures, SCs from patients #5, #6 and #10 were plated between 42 and 63 days to achieve expansion in long-term culture. **b** Immunophenotype of the cell lines was evaluated before (original cells) and at the end point of the culture according to TCRVβ2 and CD3 expression to quantify tumor cells. Inside TCRVβ2 + CD3 + population, CCR7 and CD45RO expressions were used for assessing T-cell maturation stages (naive, T_CM_, T_EM_ and T_EMRA_).

To obtain stable SC lines, PDC established for 28 days or PDX cells were cultured for an additional period until they reached more than 23 population doublings, which is the limit of division for normal CD4 + T cells [36] (Table 1). For long-term PDC, the expressions of CD3 and TCRVβ2 were stable after culture (Fig. 4a, b). According to the memory or naive T-cell phenotype, long-term PDC expanded a subpopulation already present in SCs (Fig. 4b). The majority of L5 cells showed a naive T cell phenotype expressed by only 24% of SCs in the original patient #10 sample. Similarly, the L8 cell line corresponded to the expansion of a T_EM/EMRA_ subpopulation present within the initial patient #6 sample (Fig. 4b). In the two cell lines derived from patient #5, L3 exhibited the same T_EM_ phenotype as the patient’s SCs, whereas L4 exhibited a T_EMRA_ phenotype corresponding to the loss of CD45RO and the re-expression of CD45RA by T_EM_ cells (Fig. 4b, Table 1 and data not shown). Despite this variation, this culture process provided four new SC lines (L3, L4, L5, and L8) derived from original SCs.

**Table 1:** Phenotypic characteristics and amplification properties of original patient cells and cell lines derived in vitro and/or in vivo.

Patient cells		Patient derived cell lines			
Patient number	Patient cell immunophenotype	Cell lines	Conditions to obtain cell lines	Cell line immunophenotype (major population)	Number of days to reach 23 cell divisions
Patient #2	Naive T cells/T_CM_/ T_EM_	L1	Secondary xenograft ➜culture with cytokines B	Naive T cells	132
		L2	Primary xenograft ➜ culture with cytokines B	T_CM_/T_EM_	80
Patient #5	T_EM_	L3	Culture with cytokines A	T_EM_	98
		L4	Culture with cytokines B	T_EMRA_	75
Patient #6	Naive T cells/T_CM_/ T_EM_/T_EMRA_	L8	Culture with MS5-DL1 + cytokines A	Transitory T_EM_/T_EMRA_	87
Patient #10	Naive T cells/T_CM_	L5	Culture with MS5 + cytokines B	Naive T cells	55
		L6	Primary xenograft ➜ culture with cytokines A	T_CM_/T_EM_	58
		L7	Primary xenograft ➜ culture with cytokines B	T_EM_	56

*T*_CM_ central memory T cells, *T*_EM_ effector memory T cells, *T*_EMRA_ effector memory T cells CD45RA+, ➜ followed by.

**Fig. 5:**
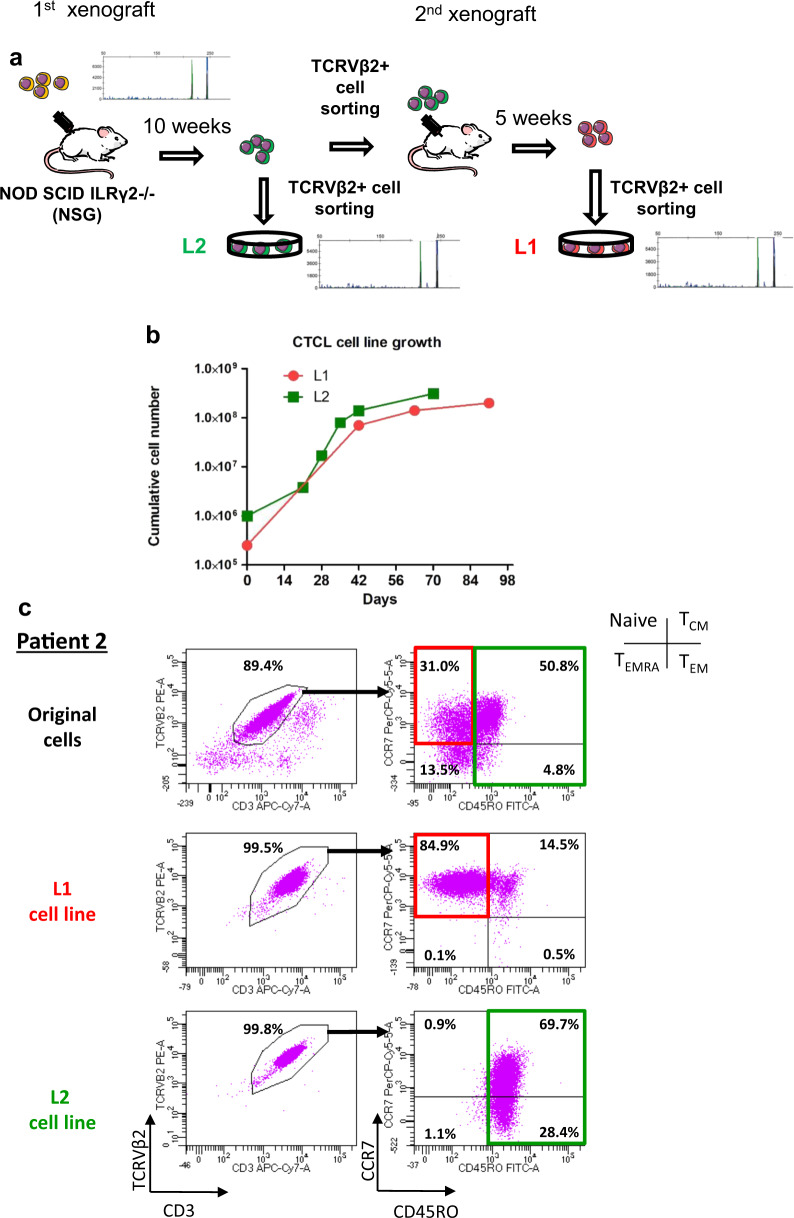
Development of different SC lines from a single patient #2 by sequential xenografting. **a** Schematic representation of L1 and L2 cell lines production from primary and secondary xenografts described in Fig. 2 and Supplementary Fig. S4. TCRγ gene rearrangements between the two PDX cell lines and original sample were determined by the Biomed-2 protocol and are shown on the diagram. **b** PDX cells after primary and secondary engraftment were cultured with cytokines mix B described in Material and methods and counted every week. Graph represents cumulative cell number along the culture. **c** Immunophenotype of L1 and L2 cell lines was evaluated before (original cells) and at the end point of the culture according to TCRVβ2 and CD3 expression to quantify tumor cells. Inside TCRVβ2 + CD3 + population, CCR7 and CD45RO expressions were used for assessing T-cell maturation stages (naive, T_CM_, T_EM_, and T_EMRA_).

To derive SC lines from PDX, involved spleens were dissected and the sorted SCs were plated in basal medium with cytokines for at least 8 weeks (Fig. 5a, b). Two L1 and L2 SC lines derived from secondary and primary xenografts of patient #2 SC reached 23 cell divisions at 132 and 80 days, respectively (Table 1). They displayed identical TCRγ monoclonal rearrangement and TCRVβ expression variants (Fig. 5a, c). However, the L1 cell line derived from naive T cells corresponded to 31% of the original SC, whereas the L2 cell line derived from the T_CM_/T_EM_ cell compartment corresponded to 55.6% of the original SC (Fig. 5c). In another patient #10, PDX-derived SC lines (L6 and L7) also underscored the presence of phenotypic variation. While the original patient #10 SC phenotype was naive T cells/T_CM_, PDX cell lines exhibited a more mature phenotype (T_CM_/T_EM_ cells) (Supplementary Fig. S2C, D).

Altogether, four additional new SC lines (L1, L2, L6, and L7) were obtained through xenografting.

### Culture and PDX models reveal clonal composition of Sézary samples

**Fig. 6:**
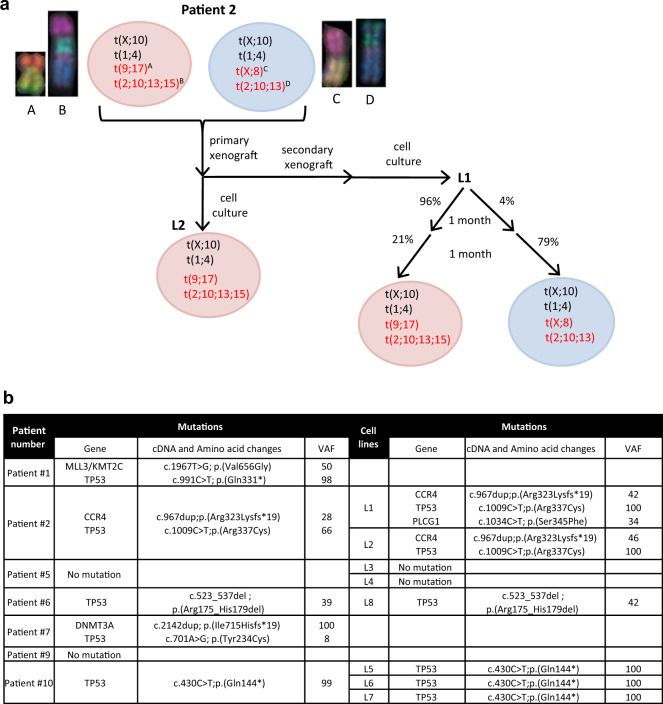
Clonal heterogeneity and evolution of SCs revealed by PDC and PDX models. **a** mFISH and aCGH analyses were performed for patient #2. Schematic representations show the clonal heterogeneity in original patient sample and after culture of PDX. Alterations mentioned in black are examples of shared alterations. The red ones are additional alterations distinguishing different sub-clones illustrated by mFISH pictures. Circles with different colors distinguish the distinct sub-clones before and after amplification. **b** Identification of genomic alterations found in original samples and after cell line expansion using a panel targeting 19 most frequently altered genes in SS. VAF: Variant allelic frequency.

To determine whether phenotypic differences before vs. after SC expansion were due to surface molecule plasticity or to a subclonal selection process, we used multicolor-fluorescence in situ hybridization (mFISH) karyotyping and aCGH analyses. For patient #2, #6, and #10, two or three major tumor subclones were detected in the original sample. Long-term culture of PDC or PDX cells achieved selection of one or two of these original subclones (Fig. 6a, Supplementary Fig. S6A–C, Supplementary Table S4), which was correlated with the above phenotypic variation (Figs. 4b, 5c). Despite the lack of original fresh metaphase cells of patient #5 SCs, aCGH parallel analyses of original SC- and PDC-derived L3 and L4 SC lines suggested that the ancestral clones acquired new alterations at chromosomes 1, 10, and 21 after expansion. Three different subclones were obtained after culture, one of them was shared by L3 and L4 in various proportions, supporting subclonal heterogeneity in this patient’s SCs (Supplementary Table S4, Supplementary Fig. S6A). For SC lines from patients #5 and #10, the few additional changes detected by mFISH analyses also suggested limited clonal evolution during amplification (Supplementary Fig. S6A, C, Supplementary Table S4). The genomic index [37] after SC amplification was close to that of original SCs, except for the L3 cell line (Supplementary Table S4). The mutational status of SC lines was evaluated *via* targeted lymphopanel analyses (Fig. 6b, Supplementary Table S5). Other than the TP53 mutation (five of seven patients), no common prevalent mutation was identified, confirming the interindividual SC genetic heterogeneity (Fig. 6b). The mutational profile of the SC lines was similar to the corresponding original SCs with a stable or enriched variant allelic frequency (VAF) of the mutations. An acquired *PLCG1* mutation detected in the L1 cell line was absent in the L2 cell line and in the original SCs (Fig. 6b).

While underscoring interindividual heterogeneity in the cell of origin and phenotype of SCs, our results support restricted intraindividual heterogeneity. PDC and PDX models and the derived SC lines revealed sub-clones already present in the original SC bulk, limited phenotypic plasticity or genomic changes after expansion.

### SC lines as a new tool for therapeutic evaluation

**Fig. 7:**
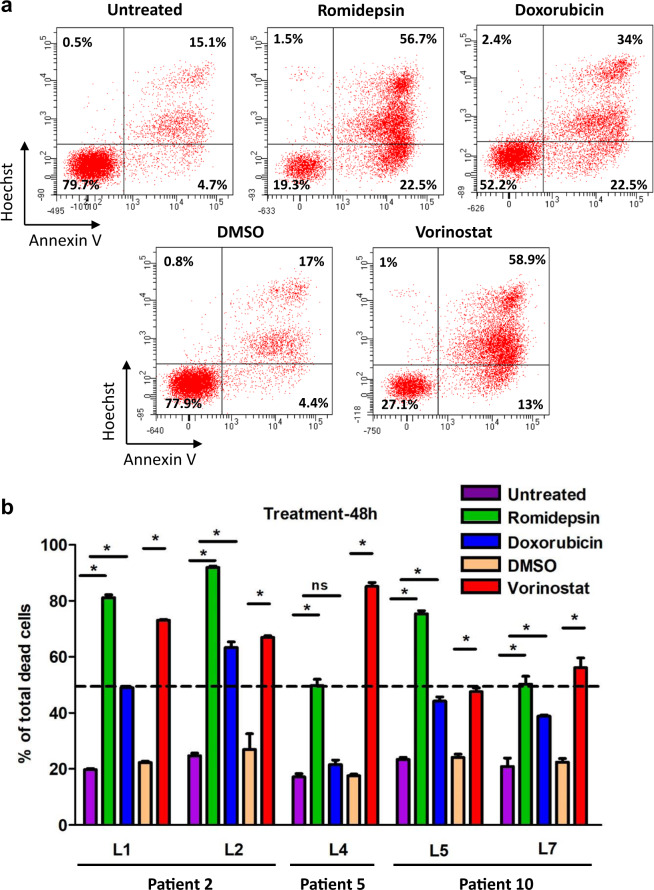
Therapeutic response to HDAC inhibitors and doxorubicin on SC lines expansion in vitro. 1 × 10^6^ cells/well were plated and treated for 48 h with romidepsin (10 nM), doxorubicin (20 nM) and vorinostat (3 µM). Untreated and DMSO conditions were added as controls. **a** Example of Annexin V+/Hoechst-, Annexin V-/Hoechst+ and Annexin V+/Hoechst+ cell proportion analysis after treatments for L1. **b** Quantification of total death in each condition for 5 SC lines (mean ± SD). Data were analyzed using Mann–Whitney test and were considered statistically significant at *P < 0.05.

To evaluate whether SC lines could be relevant for therapeutic screening, five (L1, L2, L4, L5, L7) were treated with therapies currently used to treat patients with SS (romidepsin, doxorubicin, and vorinostat) [38–40]. L1 and L2 derived from the same patient were significantly sensitive to all three treatments with slight differences in the cell death percentage (romidepsin: 81 ± 1% vs. 92 ± 0.5%; doxorubicin: 49 ± 0.6% vs. 63 ± 2% and vorinostat: 73 ± 0.3% vs. 67 ± 0.5% for L1 and L2, respectively) (Fig. 7a, b). This was also true for L5 and L7 derived from the same patient, except for romidepsin treatment (75 ± 1% vs. 50 ± 3%). Interestingly, the L4 cell line derived from another patient was resistant to doxorubicin treatment compared to other cell lines (Fig. 7b). The percentages of A+/H-, A-/H+, A+/H+ cells were evaluated for each SC line and treatment. L5 exhibited more A-/H+ cells than the other cell lines, even those derived from the same patient (L7), after romidepsin treatment (Supplementary Fig. S7A). L1 and L2 also seemed to respond differently because L2 exhibited more cells in A+/H− and less A-/H+ cells than L1, whereas there were no significant differences for A + /H + cells (Supplementary Fig. S7A–C). Altogether, our new SC lines displayed inter- and intra-individual heterogeneity in therapeutic responses, which may be used to explore the mechanisms of therapeutic resistance in patients with SS.

## Discussion

During the last several decades, very few CTCL cell lines or experimental models have been developed [17, 41, 42]. A reproducible protocol for SC expansion by patient-derived long-term culture or PDX was lacking. Here, six defined culture conditions were necessary to expand in vitro about 60% of the SS samples. A PDX model was used to directly amplify purified SCs, but with a lower success rate. Immortalizing SCs also revealed that the phenotypic and genetic diversity of SS mainly corresponded to interindividual heterogeneity according to the different cell of origin or maturation phenotype and more rarely correspond to a restricted subclonal heterogeneity already present within the bulk of some patient samples.

Standard short-term culture methods used to expand CTCL cells involve adding different cytokines such as IL-2, IL-4, IL-7, and IL-15 and unspecific mitogen as PHA in the medium. Starting from skin samples generally favors the proliferation of benign infiltrating T cells and is not suitable for growing tumor T cells [43]. In four patients, we also achieved unrelated clonal expansion when starting from unpurified blood samples despite a high content of SCs (data not shown), showing that SC sorting based on TCRVβ expression may be necessary to obtain tumor SCs from long-term cultures.

Our results also support the need to use different defined cytokines and stromal cells for SC expansion and maintenance as permissive culture conditions were patient-dependent and not predicted by the SC differentiation stage. Indeed, SCs from patient #2 and #10 displayed the same naive T cells/T_CM_ phenotype but were able to grow with or without MS5 stromal cells. In vivo, the SCs derived from these patients also showed different speeds of engraftment (9 and 18 weeks, respectively) and involved different tissues or organs depending on the patient. Recently, γ-secretase inhibitors inhibiting NOTCH signaling were found to induce apoptosis in SC lines and primary patient cells, supporting the importance of the NOTCH signaling pathway as a new target to treat patients with SS [44, 45]. In our models, coculture with MS5-DL1 overexpressing NOTCH DeltaLike 1 ligand only supported SC proliferation in patient #6 and #10 with the same efficacy than MS5 not expressing this ligand. The TCRVβ repertoire is related to the microenvironment [46] and may favor the expansion of TCRVβ2+ cells under our culture conditions, which remain to be adapted for TCRVβ2-negative SCs.

Pilot studies in immunodeficient mice have primarily used the percutaneous injection of CTCL cell lines to effectively monitor tumorigenesis at a theoretically orthotopic site [18, 21]. However, epidermotropic CTCL cells do not primarily develop in the deep dermis or hypodermis. Interestingly, one group has used an original model of fetal intrahepatic injection but its complexity and rate of animal mortality have hampered its further development [17]. To further achieve engraftment with fresh and fewer patient cells, we employed another route by engrafting SCs intrafemorally in NSG mice, because this model was shown to efficiently expand T-cell malignancy [47]. Primary PDXs were obtained in two patients with SC engraftment at the BM site, which was associated with spreading to the liver and spleen but without skin involvement, as observed when xenografting CTCL cell lines through intrahepatic injection [22]. Therefore, we performed secondary PDX at a percutaneous site and obtained both local tumorigenesis with dermal infiltration together with epidermotropism and blood dissemination, which represent a valuable model to evaluate the therapeutic response at the skin and systemic levels. Surprisingly, percutaneous xenograft of the HUT78 cell line only achieved local tumorigenesis with neither epidermotropism nor spreading capacity, supporting a difference between long-term and recently patient-derived cell lines in the expression and function of chemoreceptors and adhesion molecules.

Genetic characterization of fresh samples confirmed heterogeneity between patients and to a lesser extent within the same individual. Interestingly, original tumor cells from patient #2 contained two major subclones with naive and T_CM_/T_EM_ phenotypes that evolved independently during culture and/or xenografting providing two clonally related cell lines (L1 and L2). For patient #10, limited clonal evolution was observed after expansion in culture (loss of der(4)t(4;5) chromosome). Such intratumoral clonal heterogeneity has been reported in a transformed mycosis fungoides case after large cell transformation by comparing skin and blood compartments [48]. Therefore, preclinical models may increase our understanding of genetic homogeneity and subclonal heterogeneity starting from the patient’s sample.

The lack of preclinical models of CTCL has also limited the validation of new therapeutic agents [49, 50]. Two recent studies achieved PDX from three patients with SS using intravenous tail injection of SCs [19, 20]. These models also recapitulated the clinical and histological features of SS with blood spreading to skin and other organs such as liver and spleen providing a preclinical platform for both in vivo and in vitro screening [20]. However, no cell line has been so far maintained in long-term culture from these PDX passages. Our new SC lines with different cell differentiation stages now represent interesting tools for drug screening. For example, the two clonally related L1 and L2 cell lines derived from patient #2 exhibited the same cell death level in response to treatments. However, they displayed different proportions of A-/H+ and A+/H- cells, which could be supported by their respective naive (L1) and memory (L2) T-cell phenotype as the apoptosis regulation is different in naive and memory T cells [51]. Different sensitivities to doxorubicin were also observed between SC lines, underscoring that our new models may be used to evaluate drug response and possibly resistance mechanisms.

This study provides an original methodology to amplify SCs and obtain new SC lines, which better represent SS diversity according to cell of origin phenotype or differentiation stage. The models confirmed that SCs mostly displayed interindividual heterogeneity with no or very little plasticity in long-term expansion. Different responses to therapies may depend on the cell of origin of SCs, as well as some biological differences regarding proliferation, tumorigenesis, and migration. Using percutaneous injection, we also obtained an avatar of SS mimicking both skin and blood involvement, which may be used to test the effects of therapeutic agents at both levels, as well as the mechanisms regulating the balance between blood and skin compartments.

## Supplementary information

Supplementary Tables

Supplementary Figures

Supplementary materials and methods

## References

[1] Scarisbrick JJ, Hodak E, Bagot M, Stranzenbach R, Stadler R, Ortiz-Romero PL (2018). Blood classification and blood response criteria in mycosis fungoides and Sézary syndrome using flow cytometry: recommendations from the EORTC cutaneous lymphoma task force. Eur J Cancer.

[2] Boonk SE, Zoutman WH, Marie-Cardine A, van der Fits L, Out-Luiting JJ, Mitchell TJ (2016). Evaluation of immunophenotypic and molecular biomarkers for sézary syndrome using standard operating procedures: a multicenter study of 59 patients. J Invest Dermatol.

[3] Campbell JJ, Clark RA, Watanabe R, Kupper TS (2010). Sezary syndrome and mycosis fungoides arise from distinct T-cell subsets: a biologic rationale for their distinct clinical behaviors. Blood.

[4] Moins-Teisserenc H, Daubord M, Clave E, Douay C, Félix J, Marie-Cardine A (2015). CD158k is a reliable marker for diagnosis of Sézary syndrome and reveals an unprecedented heterogeneity of circulating malignant cells. J Invest Dermatol.

[5] Roelens M, Delord M, Ram-Wolff C, Marie-Cardine A, Alberdi A, Maki G (2017). Circulating and skin-derived Sézary cells: clonal but with phenotypic plasticity. Blood..

[6] Buus TB, Willerslev-Olsen A, Fredholm S, Blümel E, Nastasi C, Gluud M (2018). Single-cell heterogeneity in Sézary syndrome. Blood Adv.

[7] da Silva Almeida AC, Abate F, Khiabanian H, Martinez-Escala E, Guitart J, Tensen CP (2015). The mutational landscape of cutaneous T cell lymphoma and Sézary syndrome. Nat Genet.

[8] Wang L, Ni X, Covington KR, Yang BY, Shiu J, Zhang X (2015). Genomic profiling of Sézary syndrome identifies alterations of key T cell signaling and differentiation genes. Nat Genet.

[9] Chevret E, Merlio J-P (2016). Sézary syndrome: translating genetic diversity into personalized medicine. J Invest Dermatol.

[10] Borcherding N, Voigt AP, Liu V, Link BK, Zhang W, Jabbari A (2019). Single-cell profiling of cutaneous t-cell lymphoma reveals underlying heterogeneity associated with disease progression. Clin Cancer Res.

[11] Wu X, Sells RE, Hwang ST (2011). Upregulation of inflammatory cytokines and oncogenic signal pathways preceding tumor formation in a murine model of T-cell lymphoma in skin. J Invest Dermatol.

[12] Mishra A, La Perle K, Kwiatkowski S, Sullivan LA, Sams GH, Johns J (2016). Mechanism, consequences, and therapeutic targeting of abnormal IL15 signaling in cutaneous T-cell lymphoma. Cancer Discov.

[13] Kaltoft K, Bisballe S, Rasmussen HF, Thestrup-Pedersen K, Thomsen K, Sterry W (1987). A continuous T-cell line from a patient with Sézary syndrome. Arch Dermatol Res.

[14] Mann DL, O’Brien SJ, Gilbert DA, Reid Y, Popovic M, Read-Connole E (1989). Origin of the HIV-susceptible human CD4+ cell line H9. AIDS Res Hum Retroviruses.

[15] Abrams JT, Lessin S, Ghosh SK, Ju W, Vonderheid EC, Nowell P (1991). A clonal CD4-positive T-cell line established from the blood of a patient with Sézary syndrome. J Invest Dermatol.

[16] Krejsgaard T, Kopp K, Ralfkiaer E, Willumsgaard AE, Eriksen KW, Labuda T (2010). A novel xenograft model of cutaneous T-cell lymphoma. Exp Dermatol.

[17] van der Fits L, Rebel HG, Out-Luiting JJ, Pouw SM, Smit F, Vermeer KG (2012). A novel mouse model for Sézary syndrome using xenotransplantation of Sézary cells into immunodeficient RAG2(-/-) γc(-/-) mice. Exp Dermatol.

[18] Doebbeling U (2010). A mouse model for the Sézary syndrome. J Exp Clin Cancer Res.

[19] Townsend EC, Murakami MA, Christodoulou A, Christie AL, Köster J, DeSouza TA (2016). The public repository of xenografts enables discovery and randomized phase II-like trials in mice. Cancer Cell.

[20] Wu C-H, Yang C-Y, Wang L, Gao H-X, Rakhshandehroo T, Afghani S, et al. Cutaneous T cell lymphoma PDX drug screening platform identifies cooperation between inhibitions of PI3Kα/δ and HDAC. J Invest Dermatol. 2020;S0022-202X(20)31724-3. 10.1016/j.jid.2020.05.110 PMC10286854 32603749

[21] Netchiporouk E, Gantchev J, Tsang M, Thibault P, Watters AK, Hughes J-DM (2017). Analysis of CTCL cell lines reveals important differences between mycosis fungoides/Sézary syndrome vs. HTLV-1+ Leuk cell lines. Oncotarget.

[22] Andrique L, Poglio S, Prochazkova-Carlotti M, Kadin ME, Giese A, Idrissi Y (2016). Intrahepatic xenograft of cutaneous T-cell lymphoma cell lines: a useful model for rapid biological and therapeutic evaluation. Am J Pathol.

[23] van Dongen JJM, Langerak AW, Brüggemann M, Evans PaS, Hummel M, Lavender FL (2003). Design and standardization of PCR primers and protocols for detection of clonal immunoglobulin and T-cell receptor gene recombinations in suspect lymphoproliferations: report of the BIOMED-2 Concerted Action BMH4-CT98-3936. Leukemia..

[24] Armstrong F, Brunet de la Grange P, Gerby B, Rouyez M-C, Calvo J, Fontenay M (2009). NOTCH is a key regulator of human T-cell acute leukemia initiating cell activity. Blood..

[25] Kelly-Sell MJ, Kim YH, Straus S, Benoit B, Harrison C, Sutherland K (2012). The histone deacetylase inhibitor, romidepsin, suppresses cellular immune functions of cutaneous T-cell lymphoma patients. Am J Hematol.

[26] Moyal L, Feldbaum N, Goldfeiz N, Rephaeli A, Nudelman A, Weitman M (2016). The therapeutic potential of AN-7, a novel histone deacetylase inhibitor, for treatment of mycosis fungoides/sezary syndrome alone or with doxorubicin. PLoS One.

[27] Prochazkova M, Chevret E, Mainhaguiet G, Sobotka J, Vergier B, Belaud-Rotureau M-A (2007). Common chromosomal abnormalities in mycosis fungoides transformation. Genes Chromosomes Cancer.

[28] Mao X, Lillington DM, Czepulkowski B, Russell-Jones R, Young BD, Whittaker S (2003). Molecular cytogenetic characterization of Sézary syndrome. Genes Chromosomes Cancer.

[29] Verma R, Babu A. Human chromosomes: principles & techniques, 2nd ed. New York: McGraw-Hill, Inc.; 1995. 419 pp.

[30] Laharanne E, Oumouhou N, Bonnet F, Carlotti M, Gentil C, Chevret E (2010). Genome-wide analysis of cutaneous T-cell lymphomas identifies three clinically relevant classes. J Invest Dermatol.

[31] Park J, Yang J, Wenzel AT, Ramachandran A, Lee WJ, Daniels JC (2017). Genomic analysis of 220 CTCLs identifies a novel recurrent gain-of-function alteration in RLTPR (p.Q575E). Blood..

[32] Gazdar AF, Carney DN, Bunn PA, Russell EK, Jaffe ES, Schechter GP (1980). Mitogen requirements for the in vitro propagation of cutaneous T-cell lymphomas. Blood.

[33] Döbbeling U, Dummer R, Laine E, Potoczna N, Qin JZ, Burg G (1998). Interleukin-15 is an autocrine/paracrine viability factor for cutaneous T-cell lymphoma cells. Blood..

[34] Zhang Q, Nowak I, Vonderheid EC, Rook AH, Kadin ME, Nowell PC (1996). Activation of Jak/STAT proteins involved in signal transduction pathway mediated by receptor for interleukin 2 in malignant T lymphocytes derived from cutaneous anaplastic large T-cell lymphoma and Sezary syndrome. Proc Natl Acad Sci USA.

[35] Dalloul A, Laroche L, Bagot M, Mossalayi MD, Fourcade C, Thacker DJ (1992). Interleukin-7 is a growth factor for Sézary lymphoma cells. J Clin Investig.

[36] Perillo NL, Walford RL, Newman MA, Effros RB (1989). Human T lymphocytes possess a limited in vitro life span. Exp Gerontol.

[37] Lartigue L, Neuville A, Lagarde P, Brulard C, Rutkowski P, Dei Tos P (2015). Genomic index predicts clinical outcome of intermediate-risk gastrointestinal stromal tumours, providing a new inclusion criterion for imatinib adjuvant therapy. Eur J Cancer.

[38] Kogge A, Volteau C, Saint-Jean M, Peuvrel L, Brocard A, Knol A-C (2015). Vorinostat for refractory or relapsing epidermotropic T-cell lymphoma: a retrospective cohort study of 15 patients. Acta Derm Venereol.

[39] Bates SE, Eisch R, Ling A, Rosing D, Turner M, Pittaluga S (2015). Romidepsin in peripheral and cutaneous T-cell lymphoma: mechanistic implications from clinical and correlative data. Br J Haematol.

[40] Quereux G, Marques S, Nguyen J-M, Bedane C, D’incan M, Dereure O (2008). Prospective multicenter study of pegylated liposomal doxorubicin treatment in patients with advanced or refractory mycosis fungoides or Sézary syndrome. Arch Dermatol.

[41] Boudjarane J, Essaydi A, Farnault L, Popovici C, Lafage-Pochitaloff M, Beaufils N (2015). Characterization of the novel Sezary lymphoma cell line BKP1. Exp Dermatol.

[42] Nikolova M, Bagot M, Boumsell L, Bensussan A (2002). Identification of cell surface molecules characterizing human cutaneous T-cell lymphomas. Leuk Lymphoma.

[43] Harwix S, Günzl HJ, Blaschke V, Zachmann K, Neumann C (2001). Inability to culture the dominant T-cell clone from the skin of primary cutaneous T-cell lymphoma as proven by TCR gamma-chain gene sequencing. Arch Dermatol Res.

[44] Kamstrup MR, Gjerdrum LMR, Biskup E, Lauenborg BT, Ralfkiaer E, Woetmann A (2010). Notch1 as a potential therapeutic target in cutaneous T-cell lymphoma. Blood..

[45] van der Fits L, Qin Y, Out-Luiting JJ, Vermeer KG, Whittaker S, van Es JH (2012). NOTCH1 signaling as a therapeutic target in Sézary syndrome. J Invest Dermatol.

[46] Battaglia A, Ferrandina G, Buzzonetti A, Malinconico P, Legge F, Salutari V (2003). Lymphocyte populations in human lymph nodes. Alterations in CD4+ CD25+ T regulatory cell phenotype and T-cell receptor Vbeta repertoire. Immunology..

[47] Poglio S, Lewandowski D, Calvo J, Caye A, Gros A, Laharanne E (2016). Speed of leukemia development and genetic diversity in xenograft models of T cell acute lymphoblastic leukemia. Oncotarget..

[48] Prochazkova M, Chevret E, Beylot-Barry M, Vergier B, Sobotka J, Merlio J-P (2005). Large cell transformation of mycosis fungoides: tetraploidization within skin tumor large cells. Cancer Genet Cytogenet.

[49] Nicolay JP, Felcht M, Schledzewski K, Goerdt S, Géraud C (2016). Sézary syndrome: old enigmas, new targets. J Dtsch Dermatol Ges.

[50] Kohnken R, Porcu P, Mishra A (2017). Overview of the use of murine models in leukemia and lymphoma research. Front Oncol.

[51] Zhan Y, Carrington EM, Zhang Y, Heinzel S, Lew AM (2017). Life and death of activated T cells: how are they different from naïve T cells?. Front Immunol.

