# Supplementary material for: Xenograft and cell culture models of Sézary syndrome reveal cell of origin diversity and subclonal heterogeneity
Source: Leukemia. 2020 Oct 26;35(6):1696–709. doi: 10.1038/s41375-020-01068-2 (PMC8179845; doi:10.1038/s41375-020-01068-2)
Supplement: Supplementary file 1 — Supplementary Tables [file 41375_2020_1068_MOESM1_ESM.doc]

**Supplementary Tables:**

**Table S1: Patients’ characteristics.** ECP: ExtraCorporal Photopheresis, MTX: Methotrexate, TSEBT: Total Skin Electron Beam therapy. Treatments are current therapies at the time point of the analyses.

| **Patient** | **Age at diagnosis** | **Stage (diagnosis)** | **Stage (analysis)** | **Treatment (analysis)** |
| --- | --- | --- | --- | --- |
| Patient 1 | 44 | T4N0M0 B0b | T4N0M0 B2 | ECP + MTX + BEXAROTENE, VORINOSTAT |
| Patient 2 | 48 | T2N0M0 B0b | T4(T3)N+M0 B2 | CHLORAMINOPHENE |
| Patient 3 | 63 | T4N0M0 B2 | T4N0M0 B2 | ECP + BEXAROTENE + CHLORAMINOPHENE |
| Patient 4 | 66 | T4N1M0 B2 | T4N3M0 B2 | MTX, TSEBT |
| Patient 5 | 72 | T4N0M0 B2 | T4N0M0 B2 | ECP + MTX |
| Patient 6 | 77 | T2bN0M0 B1b | T4(T3)N1M0 B2 | No |
| Patient 7 | 79 | T2aN0M0 B1b | T4N0M0 B2 | GEMCITABINE, ENDOXAN |
| Patient 8 | 73 | T4N0M0 B2 | T4N0M0 B2 | ECP |
| Patient 9 | 64 | T4N0M0 B2 | T4N0M0 B2 | No |
| Patient 10 | 60 | T4N1M0 B2 | T4N1M0 B2 | No |
| Patient 11 | 77 | T4N0M0 B2 | T4N0M0 B1b | GEMCITABINE |
| Patient 12 | 57 | T4N0M0 B2 | T4N0M0 B2 | GEMCITABINE |
| Patient 13 | 76 | T4N0M0 B1b | T4N0M0 B2 | ECP + CHLORAMINOPHENE |
| Patient 22 | 87 | T4N0M0 B2 | T4N0M0 B2 | ECP + MTX |

**Table S2: Summary table of the cultures from sorted patient samples. The percentage of tumor cells in PBMC population and the cell purity after sorting are indicated. The mentioned culture is the one providing the highest proliferation rate of tumor cells.**

| **Patients** | **TCRVβ variant** | | **Tumor cell percentage in PBMC** | | **Tumor cell percentage after sorting** | | **Condition of amplification in culture for 4 weeks** | |  |
| --- | --- | --- | --- | --- | --- | --- | --- | --- | --- |
| Patient 1 | | TCRVβ22 | | 82 | | 98 | | No amplification | |
| Patient 2 | | TCRVβ2 | | 89 | | 100 | | cytokines B | |
| Patient 5 | | TCRVβ2 | | 84 | | 94 | | cytokines A | |
| Patient 6 | | TCRVβ2 | | 94 | | 100 | | MS5-DL1 + cytokines A | |
| Patient 7 | | TCRVβ13.6 | | 99 | | 100 | | No amplification | |
| Patient 9 | | TCRVβ7.1 | | 85 | | 99 | | No amplification | |
| Patient 10 | | TCRVβ2 | | 87 | | 100 | | MS5 + cytokines B | |

**Table S3. Patient-derived xenograft (PDX) for 14 samples. IF:** intrafemoral, **PC:** percutaneous

| **Patient** | **Cell number / mouse** | **Transplant** | **Site of injection** | **Positive mice number / total mice injected** |
| --- | --- | --- | --- | --- |
| Patient 1 | 1x10^6^ | primary | IF | 0 / 10 |
| Patient 2 | 1x10^6^ | primary | IF | 4 / 5 |
|  | 1x10^6^ | secondary | IF | 5 / 5 |
|  | 0.4-2x10^6^ | secondary | PC | 7 / 8 |
| Patient 3 | 1x10^6^ | primary | IF | 0 / 5 |
| Patient 4 | 1x10^6^ | primary | IF | 0 / 10 |
| Patient 5 | 1x10^6^ | primary | IF | 0 /5 |
| Patient 6 | 1x10^6^ | primary | IF | 0 / 5 |
| Patient 7 | 1x10^6^ | primary | IF | 0 / 5 |
| Patient 8 | 1x10^6^ | primary | IF | 0 / 5 |
| Patient 9 | 1x10^6^ | primary | IF | 0 / 5 |
| Patient 10 | 1x10^6^ | primary | IF | 3 / 5 |
|  | 3x10^5^ | secondary | IF | 4 / 5 |
| Patient 11 | 1x10^6^ | primary | IF | 0 / 5 |
| Patient 12 | 1x10^6^ | primary | IF | 0 / 10 |
| Patient 13 | 1x10^6^ | primary | IF | 0 / 10 |
| Patient 22 | 1x10^6^ | primary | IF | 0 / 10 |

**Table S4: Multicolor-FISH and aCGH analyses of fresh patient’s cells and new SC lines. Bold type: differential genomic alterations. GI: Genomic index. NA : Not Available**

| **Sample** | **Karyotype (on the basis of mFISH results)** | **aCGH (Mb)** | **GI** |
| --- | --- | --- | --- |
| **Patient 2** | 45~48,X,t(X;10),del(1)(q3?),del(1)(q11),  der(2)(22q?->22q?::2p1?->2q?::1q?),der(2)t(2;10)(q?;p?),add(3)(qter),  der(6)t(6;11)(q?;q?),+der(7)t(7;13)(?;q?),+der(7)t(7;11)(?;?),  der(8)t(8;14)(q?;q?),-9,der(9)t(2;9)(?;p?),der(10)(15q?::10::13q?::10::2),  der(11)(6::11::5), del(13)(q?),der(13)t(11;13)(?;q?),-14,-15,  der(16)t(16;17)(p?;?),der(17)t(9;17)(?;p?),+del(17)(?),  der(18)t(11;18)(?;?), der(18)t(16;18)(?;?),der(19)t(6;19)(?;?),  der(21)t(10;21)(?;?),der(22)(22q?::2::6),der(22)t(1;22)(?;p?),  +mar1~4[cp9] /  45~47,der(X)t(X;8)(?;?),t(X;10)(q?;q?),del(1)(q3?),del(1)(q11),  der(2)(22q?->22q?::2p1?->2q?::1q?),der(2)t(2;10)(q?;p?),add(3)(qter),  der(6)t(6;11)(q?;q?),+iso(7)(q10),+der(7)t(7;11)(?;?),-8, der(8)t(8;14)(q?;q?),der(9)t(2;9)(?;p?),  der(10)(10p?->10q::13q?::10q::13q?::2?),-11,der(11)t(6;11)(q?;?), del(13)(q?),der(13)t(11;13)(?;q?),-14,der(16)t(16;17)(p?;?),  der(18)t(11;18)(?;?),der(18)t(16;18)(?;?),der(19)t(6;19)(?;?),  der(22)(22q::2::6),der(22)t(1;22)(?;p?),+mar1~2[cp9] | arr**1p36.33-p36.11(1,15-23,34)x3**,1p13.3 - p13.2(111,55-112,03)x1,  2q12.3(109,23-109,46)x1,2q13(111,6-111,9)x1,  2q37.1-q37.3(235,11-243,04)x0,4q31.3(152,61-153,84)x0,  **5p15.33-p14.1(-)x3**,6p21.31-p11.2(35,42-57,36)x3,  **6q11.1-q15(62,30-90,87)x3**,6q15-q16.3(90,91-100,79)x1,  6q16.3-q27(100,81-170,87)x3,(7)x3,8p23.3-p12(0,31-36,43)x1,  8q13.1-q24.21(66,11-128,98)x3,9p21.3(21,80-22,21)x0,  10p12.31-p12.1(21,92-28,90)x1,10p11.23-p11.22(30,20-35,60)x1,  10q21.2-q21.3(63,52-65,36)x1,10q23.3 -q25.1(96,87-110,04)x1,  11p15.4-p15.3(7,34-12,68)x3,12q13.11(48,04-48,49)x1,  13q14.2-q14.3(50,14-50,97)x1,**14q12-q21.3(25,46-50,57)x3**,  **14q23.1-q32.11(59,10-90,36)x3,14q32.13-q32.33(96,17-107,22)x3,**  16q22.1(67,60-67,80)x1,17p13.1(75,30-80,47)x1,  17q11.2-q25.3(31,20-81,07)x3,19p13.3-p13.12(0,57-14,49)x3,  19p13.12(145,17-145,72)x1,22q12.1-q12.2(28,87-32,09)x1,  22q13.33(50,53-51,15)x1 | 60 |
| **L1** | 45~48,X,t(X;10),del(1)(q3?),del(1)(q11),  der(2)(22q?->22q?::2p1?->2q?::1q?),der(2)t(2;10)(q?;p?),add(3)(qter),  der(6)t(6;11)(q?;q?),+der(7)t(7;13)(?;q?),+der(7)t(7;11)(?;?),  der(8)t(8;14)(q?;q?),-9,der(9)t(2;9)(?;p?),der(10)(15q?::10::13q?::10::2),  der(11)(6::11::5), del(13)(q?),der(13)t(11;13)(?;q?),-14,-15,  der(16)t(16;17)(p?;?),der(17)t(9;17)(?;p?),+del(17)(?),  der(18)t(11;18)(?;?),der(18)t(16;18)(?;?),der(19)t(6;19)(?;?),  der(21)t(10;21)(?;?),der(22)(22q?::2::6),der(22)t(1;22)(?;p?),  +mar1~4[cp3] /  45~47,der(X)t(X;8)(?;?),t(X;10)(q?;q?),del(1)(q3?),del(1)(q11),  der(2)(22q?->22q?::2p1?->2q?::1q?),der(2)t(2;10)(q?;p?),add(3)(qter),  der(6)t(6;11)(q?;q?),+iso(7)(q10),+der(7)t(7;11)(?;?),-8, der(8)t(8;14)(q?;q?),der(9)t(2;9)(?;p?),  der(10)(10p?->10q::13q?::10q::13q?::2?),-11,der(11)t(6;11)(q?;?), del(13)(q?),der(13)t(11;13)(?;q?),-14,der(16)t(16;17)(p?;?),  der(18)t(11;18)(?;?),der(18)t(16;18)(?;?),der(19)t(6;19)(?;?),  der(22)(22q::2::6),der(22)t(1;22)(?;p?),+mar1~2[cp3] | arr**1p36.33-p36.11(1,15-23,34)x3**,1p13.3 - p13.2(111,55-112,03)x1, 2q12.3(109,23-109,46)x1,2q13(111,6-111,9)x1,  2q37.1-q37.3(235,11-243,04)x0,4q31.3(152,61-153,84)x0,  6p21.31-p11.2(35,42-57,36)x3,6q15-q16.3(90,91-100,79)x1,  **6q21(106,48-112,94)x1**,**7p22.3-p13(0,08-44,50)x3,**  **7q11.21-q36.3(62,46-159,08)x4**,**8p23.3-p12(0,24-29,87)x1**,  9p21.3(21,80-22,21)x0,10p12.31-p12.1(21,92-28,90)x1,  10p11.23-p11.22(30,20-35,60)x1,10q21.2-q21.3(63,52-65,36)x1,  10q23.3 -q25.1(96,87-110,04)x1,11p15.4-p15.3(7,34-12,68)x3, 12q13.11(48,04-48,49)x1,13q14.2-q14.3(50,14-50,97)x1,  **13q14.3-q34(51,51-115,09)x1**,**16p13.3-p13.12(0,10-14,16)x1**,  16q22.1(67,60-67,80)x1,17p13.1(75,30-80,47)x1,  17q11.2-q25.3(31,20-81,07)x3,19p13.3-p13.12(0,57-14,49)x3,  19p13.12(145,17-145,72)x1,22q12.1-q12.2(28,87-32,09)x1,  22q13.33(50,53-51,15)x1,**Xp22.33-p11.22(3,95-52,06)x1** | 56 |
| **L2** | 45~48,X,t(X;10),del(1)(q3?),del(1)(q11),  der(2)(22q?->22q?::2p1?->2q?::1q?),der(2)t(2;10)(q?;p?),add(3)(qter),  der(6)t(6;11)(q?;q?),+der(7)t(7;13)(?;q?),+der(7)t(7;11)(?;?),  der(8)t(8;14)(q?;q?),-9,der(9)t(2;9)(?;p?),der(10)(15q?::10::13q?::10::2),  der(11)(6::11::5), del(13)(q?),der(13)t(11;13)(?;q?),-14,-15,  der(16)t(16;17)(p?;?),der(17)t(9;17)(?;p?),+del(17)(?), der(18)t(11;18)(?;?),der(18)t(16;18)(?;?),der(19)t(6;19)(?;?),  der(21)t(10;21)(?;?),der(22)(22q?::2::6),der(22)t(1;22)(?;p?),  +mar1~4[cp10] | arr**1p22.1-p13.2(94,01-112,21)x1**,1p13.3-p13.2(111,55-112,03)x1, 2q12.3(109,23-109,46)x1,2q13(111,6-111,9)x1,  2q37.1-q37.3(235,11-243,04)x0,4q31.3(152,61-153,84)x0,  **5p15.33-p14.1(-)x3**,6p21.31-p11.2(35,42-57,36)x3,  **6q11.1-q15(62,30-90,87)x3**,6q15-q16.3(90,91-100,79)x1,  6q16.3-q27(100,81-170,87)x3,(7p)x3,  **7q11.21-q36.1(62,55-149,18)x3**, 9p21.3(21,80-22,21)x0,  **9q13-q32(66,50-115,56)x1,9q33.1-q34.3(118,20-141,01)x3**, 10p12.31-p12.1(21,92-28,90)x1,10p11.23-p11.22(30,20-35,60)x1, 10q21.2-q21.3(63,52-65,36)x1,10q23.3-q25.1(96,87-110,04)x1, 11p15.4-p15.3(7,34-12,68)x3,12q13.11(48,04-48,49)x1,  13q14.2-q14.3(50,14-50,97)x1,**15q11.1-q13.3(20,10-31,42)x1**,  16q22.1(67,60-67,80)x1,17p13.1(75,30-80,47)x1,  **17p13.1-p12(9,23-14,14)x1,**17q11.2-q25.3(31,20-81,07)x3,  19p13.3-p13.12(0,57-14,49)x3,19p13.12(145,17-145,72)x1,  22q12.1-q12.2(28,87-32,09)x1,22q13.33(50,53-51,15)x1 | 64 |
| **Patient 5** | NA | arr1p36.12-p36.11(23,37-24,25)x1,1p36.11-p35.3(25,85-28,08)x1, 1p35.1-p34.3(32,48-38,03)x1,1p31.3(70,58-79,18)x1,  2p23.3(25,35-26,42)x1, 2p22.2(26,10-28)x1,  4q31.3(153,16-154,62)x1,5q31.1-q31.2(131,81-139,15)x3,  5q33.1-5qter(150,71-180,69)x3,6p25.3(0,26-0,37)x1,(8)x3,  9p21.3(21,39-22,46)x0,10p15.3-p12.1(0,13-24,8)x3,  11p13(32,8-35,4)x0,11q22.3(107,19-108,79)x0,  14q32.33(106,33-106,53)x0,16q21-q22.1(63,25-69,20)x0,(17p)x1,  (17q)x3,(18p)x1,(18q)x3,(19p13.3)x1 | 35 |
| **L3** | 48,XX,add(1)(p?),der(1)t(1;12)(p1?;p3?),der(5)add(5)(p?)t(5;16)(q?;q?),  inv(6)(p?;q?),+8,der(12)t(1;12)(p?;p?),der(16)t(5;16)(q?;q?),  der(17)i(17)(q10)t(2;17)(p?;q?),der(18)t(10;18)(p?;p?),+der(18)t(10;18),  idic(21)(q)[cp12] /46,X,der(X)t(X;1)(q?;q?),add(1)(p?),der(1)t(1;12)(p1?;p3?),  der(5)add(5)(p?)t(5;16)(q?;q?),inv(6)(p;q),-10,der(12)t(1;12)(p1?;p3?),  der(16)t(5;16)(q?;q?),der(17)i(17)(q10)t(2;17)(p?;q?),  der(18)t(10;18)(p?;p?),+der(18)t(10;18),add(21)(q?)[cp3] | arr1p36.12-p36.11(23,37-24,25)x1,**1p36x3,**  1p36.11-p35.3(25,85-28,08)x1,1p35.1- p34.3(32,48-38,03)x1,  **1p33-32x3,**1p31.3(70,58-79,18)x1,2p23.3(25,35-26,42)x1, 2p22.2(26,10-28)x1,4q31.3(153,16-154,62)x1,  5q31.1-q31.2(131,81-139,15)x3,5q33.1-5qter(150,71-180,69)x3,  6p25.3(0,26-0,37)x1,(8)x3,9p21.3(21,39-22,46)x0,  10p15,3-p12.1(0,13-24,8)x3,**10p12.1-10qterx1**,  11p13(32,8-35,4)x0,11q22.3(107,19-108,79)x0,  14q32.33(106,33-106,53)x0,16q21-q22.1(63,25-69,20)x0,  (17p)x1,(17q)x3,(18p)x1,(18q)x3,(19p13.3)x1**, 21qx3, Xq25-qterx1** | 46 |
| **L4** | 48~49,XX,add(1)(p?),der(1)t(1;12)(p1?;p3?),  der(5)add(5)(p?)t(5;16)(q?;q?),inv(6)(p?;q?),+8,der(12)t(1;12)(p1?;p3?), der(16)t(5;16)(q?;q?),der(17)i(17)(q10)t(2;17)(p?;q?), der(18)t(10;18)(p?;p?),+der(18)t(10;18),add(21)(q?),+add(21)(q?)[cp13] | arr1p36.12-p36.11(23,37-24,25)x1,**1p36x3,**  1p36.11-p35.3(25,85-28,08)x1,1p35.1-p34.3(32,48-38,03)x1,  **1p33-32x3,**1p31.3(70,58-79,18)x1,2p23.3(25,35-26,42)x1, 2p22.2(26,10-28)x1,4q31.3(153,16-154,62)x1,  5q31.1-q31.2(131,81-139,15)x3,5q33.1-5qter(150,71-180,69)x3,  6p25.3(0,26-0,37)x1,(8)x3,9p21.3(21,39-22,46)x0,  10p15.3-p12.1(0,13-24,8)x3,11p13(32,8-35,4)x0,  11q22.3(107,19-108,79)x0,14q32.33(106,33-106,53)x0,  16q21-q22.1(63,25-69,20)x0,(17p)x1,(17q)x3,(18p)x1,(18q)x3,  (19p13.3)x1**,21qx3** | 38 |
| **Patient 10** | 48,XX,der(2)t(2;4)(p?;p?),add(3)(q?),+4,der(4)t(2;4)(p?;p?),  t(4;15)(q?;q?),der(5)t(5;9)(q?;p?),+8,der(9)t(5;9)t(5;12),  der(12)t(5;12)(q?;p?),t(13;14)(q?;q?),der(16)t(2;16)(?;q?),add(17)(q?),  add(20)(q?)[cp8]  /49,sl,+5[cp16] | arr1p36.1(27,10-27,32)x1,2p21-p13.6(47,67-48,73)x1,  3q24-q25.1(147,6-149,2)x0,3q25.1-q25.31(149-156)x4,  3q25.31-q25.33(153-155)x0,3q26.1-3q26.31(160,73-172,47)x4, 3q26.31-3q26.33(173,02-182,58)x0,  3q26.33-q27.2(182,63-185,39)x3,3q27.2-3q29(185,69-195)x0,  3q29(195,05-197,83)x3,(4)x3,(5)x3,6p21.31-p21.2(36,22-36,71)x0,  (8)x3,9p21.3(21,85-23,08)x0,10p14(7,95-9,28)x0,  12p13.2-p13.1(12,31-13,25)x0,12q21.33-q22(92,02-95,66)x0,  14q12-q21.1(29,71-39,71)x0,14q22.1(52,45-53,90)x0,  15q24.2(75,66-76,19)x0,16q24.2-q24.3(88,68-89,29)x1,  17p13.3-p11.2(0,05-18,92)x1,17p12-q22(19,14-53,02)x3,  17q22(50,82-52,96)x1,17q22-q24.3(54,37-64,32)x3,  17q24.3(67,36-70,28)x1,17q24.3-q25.3(70,28-81,09)x3 | 60 |
| **L5** | 48,XX,der(2)t(2;4)(p?;p?),add(3)(q?),+4,der(4)t(2;4),t(4;15)(q?;q?),  der(5)t(5;9)(q?;p?),+8,der(9)t(5;9)t(5;12),der(12)t(5;12)(q?;p?),  t(13;14)(q?;q?),der(15)t(4;15)(q?;q?),der(16)t(2;16)(?;q?),add(17)(q?),  add(20)(q?)[2] /  49,sl,+5[1] | NA |  |
| **L7** | 47,XX,der(2)t(2;4)(p?;p?),add(3)(q?),der(5)t(5;9)(q?;p?),+8,  der(9)t(5;9)t(5;12),der(12)t(5;12)(q?;p?),t(13;14)(q?;q?),  der(15)t(4;15)(q?;q?),der(16)t(2;16)(?;q?),  add(17)(q?),add(20)(q?)[1]  /48,sl,+5[13] | arr1p36.1(27,10-27,32)x1,2p21-p13.6(47,67-48,73)x1,  **2q33.1(200-201)x0,2q33.1-2q33.3(201,2-205,9)x4**,  3q24-q25.1(147,6-149,2)x0,3q25.1-q25.31(149-156)x4,  3q25.31-q25.33(153-155)x0,3q26.1-3q26.31(160,73-172,47)x4, 3q26.31-3q26.33(173,02-182,58)x0,  3q26.33-q27.2(182,63-185,39)x3,3q27.2-3q29(185,69-195)x0,  3q29(195,05-197,83)x3,**4q25-qterx3**,(5)x3,  6p21.31-p21.2(36,22-36,71)x0,(8)x3,9p21.3(21,85-23,08)x0, 10p14(7,95-9,28)x0,12p13.2-p13.1(12,31-13,25)x0,  12q21.33-q22(92,02-95,66)x0,14q12-q21.1(29,71-39,71)x0,  14q22.1(52,45-53,90)x0,**15q24.2-q26.3(75,66-101,85)x0**,  16q24.2-q24.3(88,68-89,29)x1,17p13.3-p11.2(0,05-18,92)x1,  17p12-q22(19,14-53,02)x3,17q22(50,82-52,96)x1,  17q22-q24.3(54,37-64,32)x3,17q24.3(67,36-70,28)x1,  17q24.3-q25.3(70,28-81,09)x3 | 64 |
| **Patient 6** | 44,X,der(X)t(X;22),add(1)(q?),add(3)(p?),der(4)t(1;4)(?;p?),t(5;7)(q?;p?),+7,t(7;8)(q?;q?),der(9)t(9;13)(?;?),der(9)t(9;15)(?;?),10,  der(12)t(1;12)(?;pter),-13,-15,der(16)(8::10::16)(?;?;?),  der(17)t(X;17)(?;p?),der(17)t(10;17)(q?;qter),  +der(17)t(10;17),der(20)t(10;20)(?;p?),del(22)(q?)[12]  /46,X,add(1)(q?),add(3)(p?),der(4)t(1;4)(?;p?),t(5;7)(q?;p?),+7,  t(7;8)(q?;q?),der(9)t(9;13)(?;?),+der(9)t(9;13)(?;?),der(9)t(9;15)(?;?),-10, -13,-15,der(17)t(X;17)(?;p?),der(17)t(10;17)(q?;qter),+der(17)t(10;17),  der(20)t(10;20)(?;p?),del(22)(q?)[2]  /46,XX[2] | NA |  |
| **L8** | 44,X,der(X)t(X;22),add(1)(q?),add(3)(p?),der(4)t(1;4)(?;p?),t(5;7)(q?;p?),+7,t(7;8)(q?;q?),der(9)t(9;13)(?;?),der(9)t(9;15)(?;?),-10, der(12)t(1;12)(?;pter),-13,-15,der(16)(8::10::16)(?;?;?),  der(17)t(X;17)(?;p?),der(17)t(10;17)(q?;qter),+der(17)t(10;17),  der(20)t(10;20)(?;p?),del(22)(q?)[25] | NA |  |

**Table S5: Genes included in the lymphopanel analysis.**

| **Gene** | **Transcript Reference** | **Design: Targeted Exons or whole coding sequence** | **Chromosome location** |
| --- | --- | --- | --- |
| *ARID1A* | NM_006015 | All exons | 1p36.11 |
| *CARD11* | NM_032415 | All exons | 7p22.2 |
| *CCR4* | NM_005508 | Exon 2 | 3p22.3 |
| *CD28* | NM_006139 | All exons | 2q33.2 |
| *DNMT3A* | NM_022552 | Exons 3, 7, 9-11, 13-23 | 2p23.3 |
| *FAS* | NM_000043 | All exons | 10q23.31 |
| *FASN* | NM_004104 | Exons 33 & 34 | 10q24.1 |
| *IDH2* | NM_002168 | Exon 4 | 15q26.1 |
| *JAK3* | NM_000215 | All exons | 19p13.11 |
| *KDM6B* | NM_001080424 | All exons | 17p13.1 |
| *MLL3/KMT2C* | NM_170606 | All exons | 7q36.1 |
| *PLCG1* | NM_002660 | All exons | 20q12 |
| *RHOA* | NM_001664 | Exons 2 & 4 | 3p21.31 |
| *SETD1B* | NM_015048 | All exons | 12q24.31 |
| *STAT3* | NM_139267 | Exons 19-21 | 17q21.2 |
| *STAT5B* | NM_1012448 | Exons 14-17 | 17q21.2 |
| *TET2* | NM_001127208 | All exons | 4q24 |
| *TP53* | NM_000546 | All exons | 17p13.1 |
| *ZEB1* | NM_001174096 | All exons | 10p11.22 |
