# Supplementary material for: Xenograft and cell culture models of Sézary syndrome reveal cell of origin diversity and subclonal heterogeneity
Source: Leukemia. 2020 Oct 26;35(6):1696–709. doi: 10.1038/s41375-020-01068-2 (PMC8179845; doi:10.1038/s41375-020-01068-2)
Supplement: Supplementary file 2 — Supplementary Figures [file 41375_2020_1068_MOESM2_ESM.pdf]

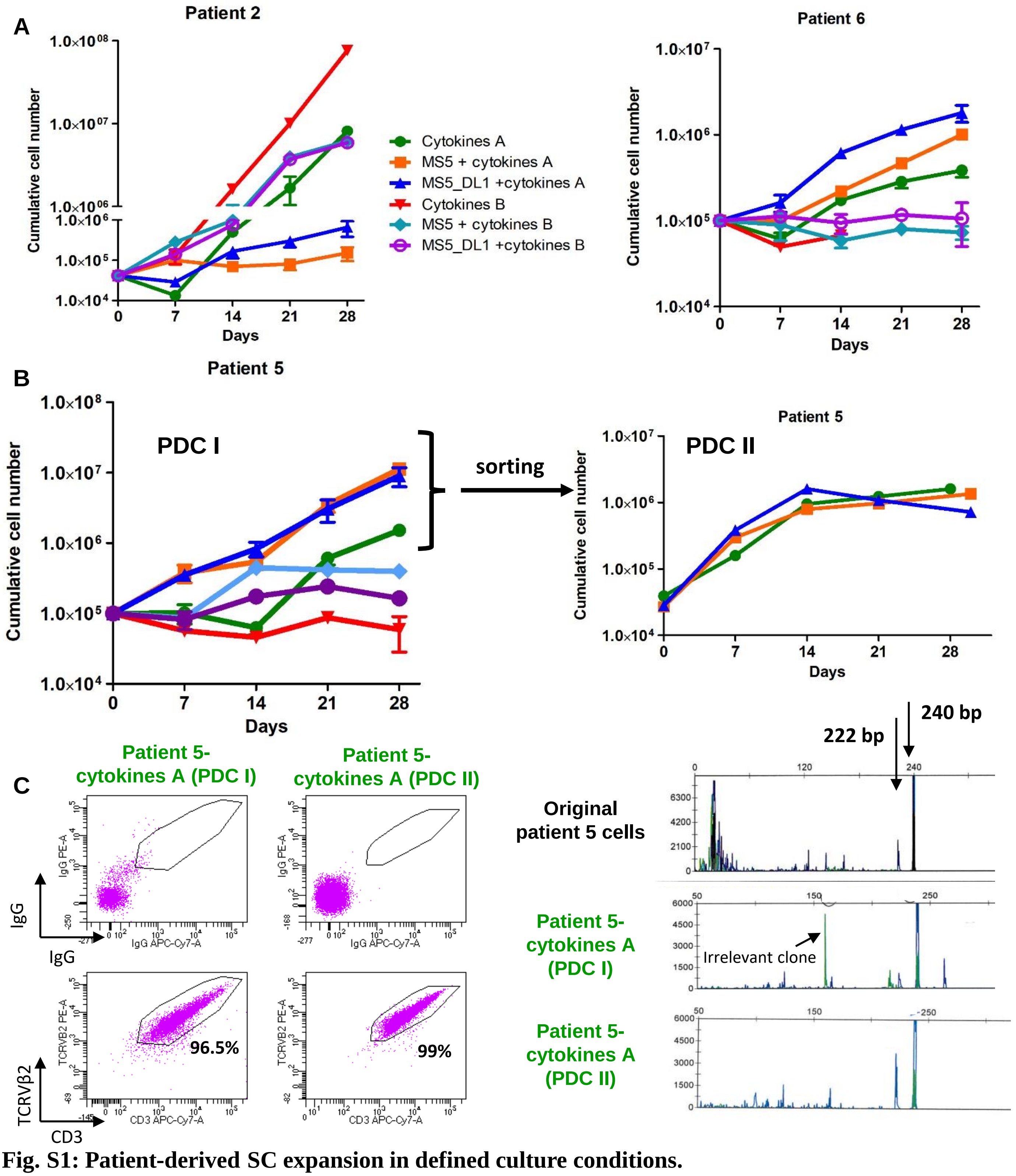

Fig. S1: Patient-derived SC expansion in defined culture conditions.

(**A**) Primary culture of patient #2 and #6 SCs for 28 days after tumor cell sorting. Cells were plated on 6 distinct culture conditions and counted every week. (**B**) Patient #5 cells were plated for primary culture (PDC I) but a mix of tumor and irrelevant clones were expanded. Then, a secondary culture (PDC II) of sorted TCRVβ2+CD3+CD4+CD8- PDC I tumor cells was performed to amplify pure tumor cell population. Cells were plated on 3 distinct culture conditions and counted every week. Graph represents the results for 3 conditions: cultures in basal medium + cytokines A (i) , co-culture on MS5 (ii) or MS5-DL1 + cytokines A (iii). Only cells expanded in (i) are pure tumor cells as shown in (**C**) by the detection of TCRVβ2+CD3+ cells by FACS (*left panel*) and by PCR on TCRγ locus (*right panel*).

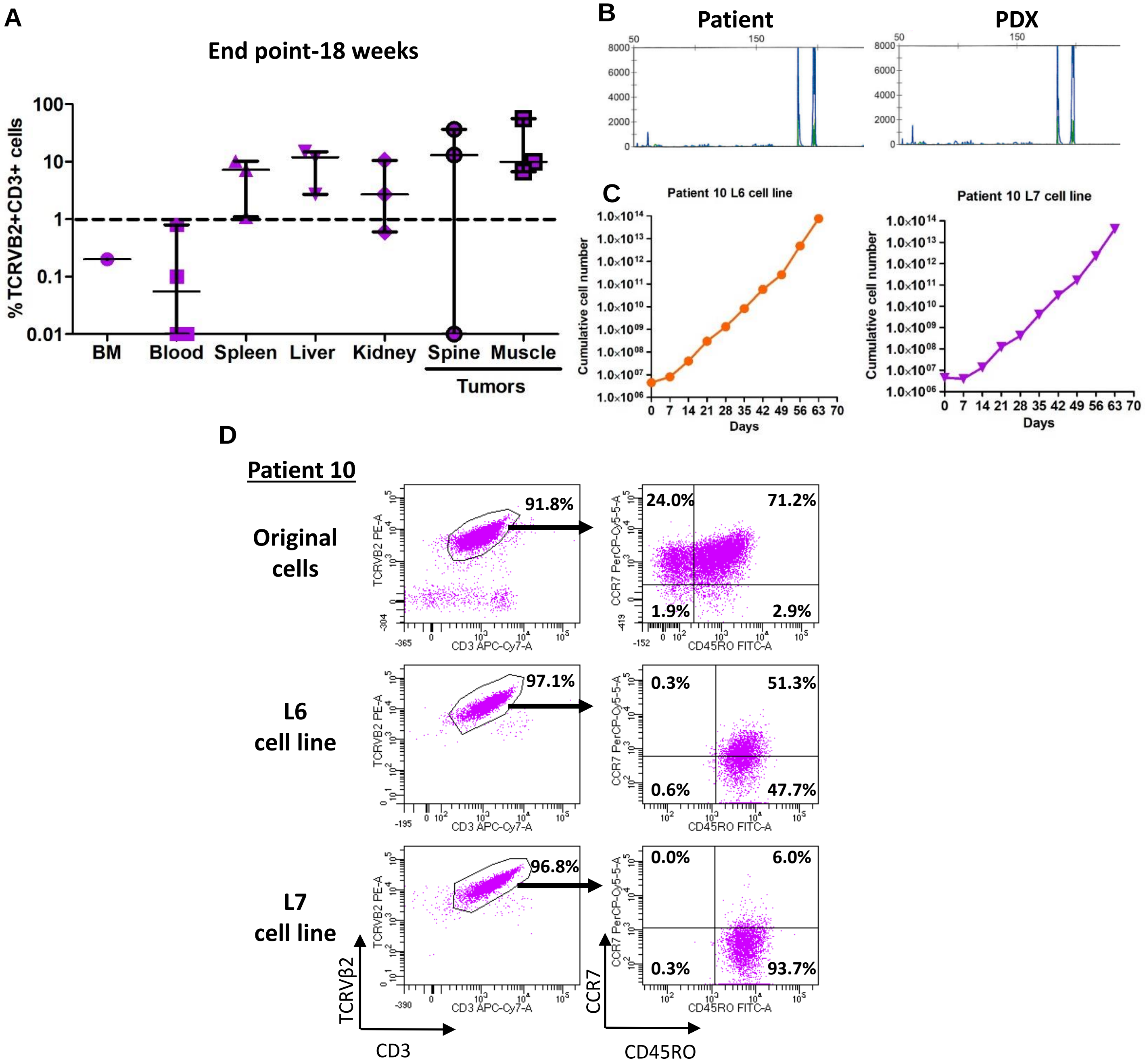

Fig. S2: Patient #10 SC xenograft in immunodeficient NSG mice and generation of new SC lines from xenografted cells. (**A**) 1x10^6 tumor cells (TCRVβ2+CD3+CD4+CD8-) from patient #10 were injected in NSG mice. Mice were sacrificed 18 weeks after transplant when mice showed signs of illness or percentage of SCs in BM was at least 50%. Cell suspensions were obtained from different tissues and analyzed for TCRVβ2 and CD3 expression by FACS. (**B**) Same monoclonal TCRγ locus rearrangement between patient SCs and PDX was detected**.** (**C**) Long-term expansion of primary xenograft cells by defined culture conditions during 63 days. L6 and L7 cell lines derived from culture in basal medium implemented with cytokines A or B respectively. (**D**) Immunophenotype of SCs before (original cells) and at long-term culture end point according to TCRVβ2 and CD3 expression. Inside TCRVβ2+CD3+ population, CCR7 and CD45RO expression was analyzed to determine their T-cell maturation stage (naive, T_{CM}, T_{EM} and T_{EMRA}).

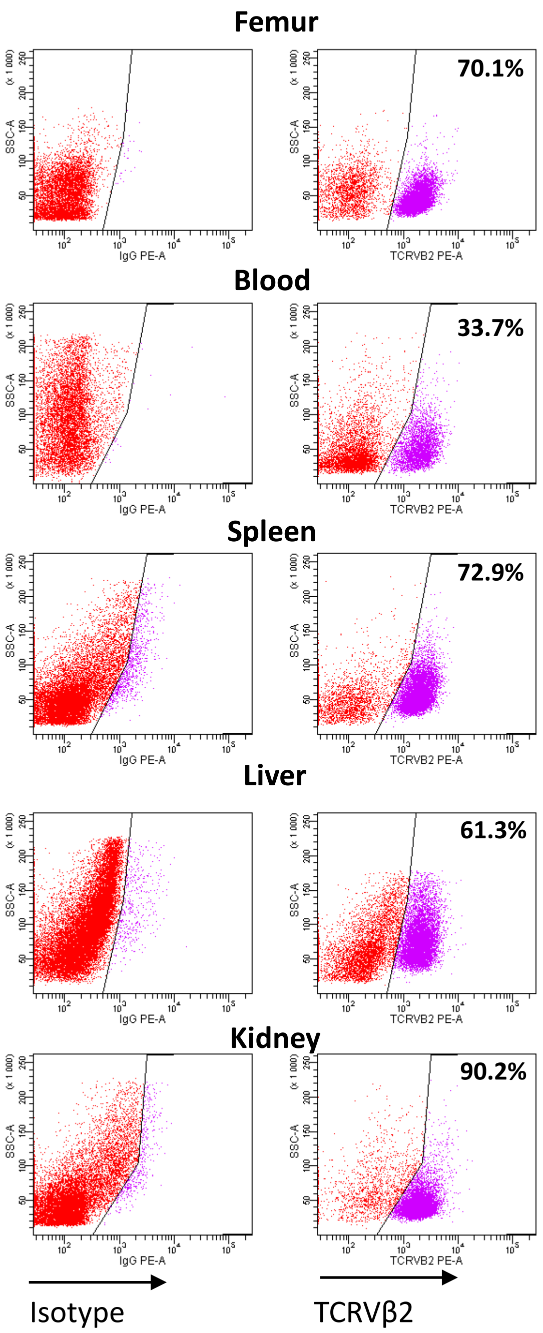

Fig. S3: Patient#2 SCs expansion in immunodeficient NSG mice. 1x10^6 patient#2 SCs (TCRVβ2+CD3+CD4+CD8-) were injected in NOD SCID IL2Rγc-/- (NSG) immunodeficient mice. At sacrifice, cell suspensions from different tissues were prepared and TCRVβ2 expression analyzed by FACS. Dot plots represent the percentage of TCRVβ2+ cells in femur, blood, spleen, liver and kidney and their respective isotype control.

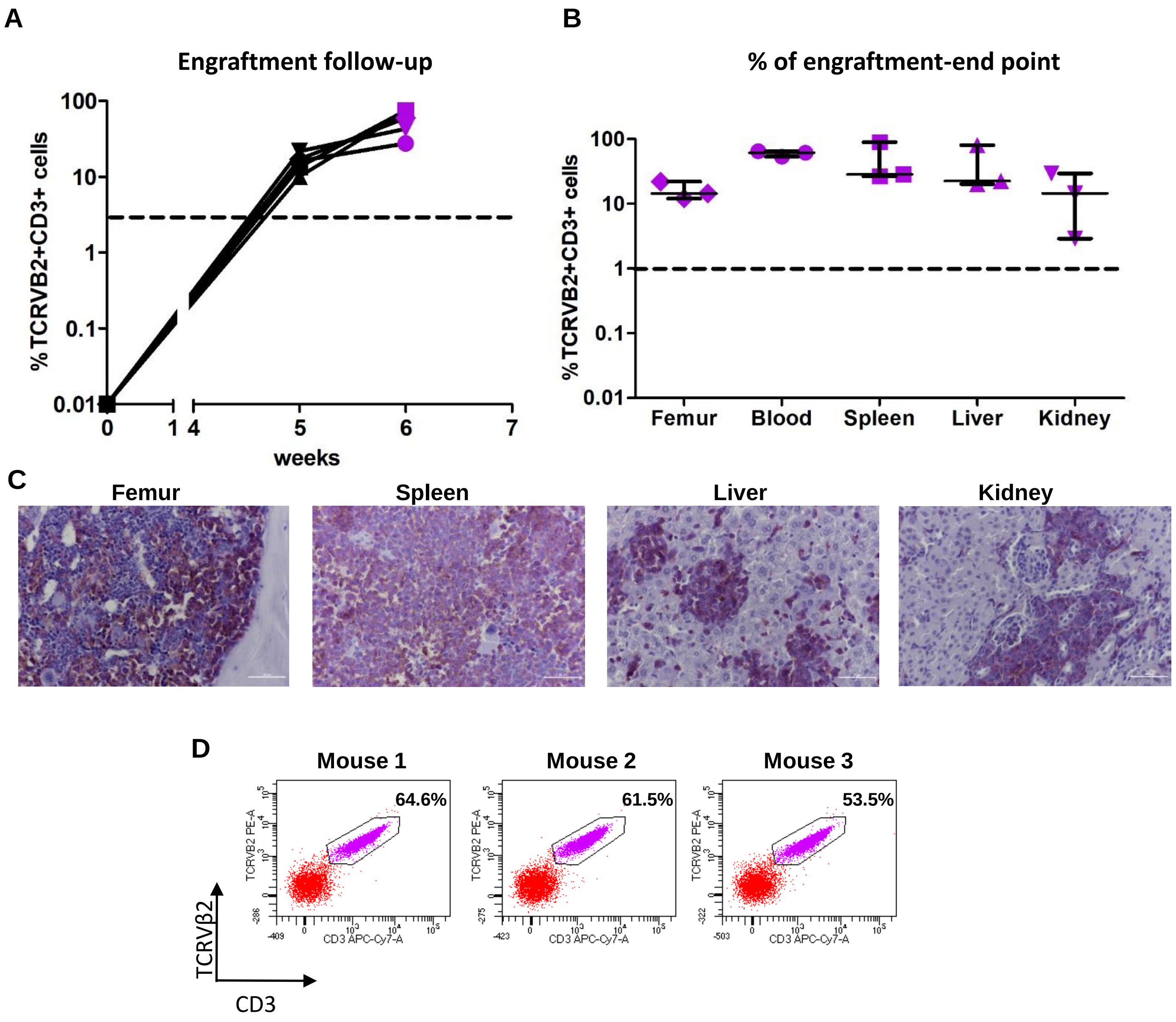

Fig. S4: Renewal of patient SCs in secondary transplant at either bone marrow or skin site.
(**A**) 1x10^{6} tumor cells (TCRVβ2+CD3+CD4+CD8-) from patient #2 derived from primary xenograft were injected intrafemorally in secondary NOD SCID IL2Rγc-/- immunodeficient (NSG) mice and percentage of tumor cells was detected in BM by FACS from week-5 and sacrificed 1 week later. Purple dots represent the percentage of engraftment in femur at sacrifice. (**B**) Mice were sacrificed when percentage of SCs in BM reached at least 50% or when mice shown signs of illness. The cells from different tissues were prepared and percentage of TCRVβ2+CD3+ cells analyzed by FACS. Graph represents the percentage of TCRVβ2+CD3+ cells in blood, spleen, liver and kidney. For each tissue, median and range are shown. (**C**) Identification of human cells by HLA-ABC immunostaining in femur, spleen, liver and kidney sections. (**D**) 0.4-2x10^{6} tumor cells (TCRVβ2+CD3+CD4+CD8-) from patient #2 derived from primary xenograft were injected percutaneously in secondary NSG mice. Dot plots representing the percentage of circulating SCs (TCRVβ2+CD3+) detected by FACS.

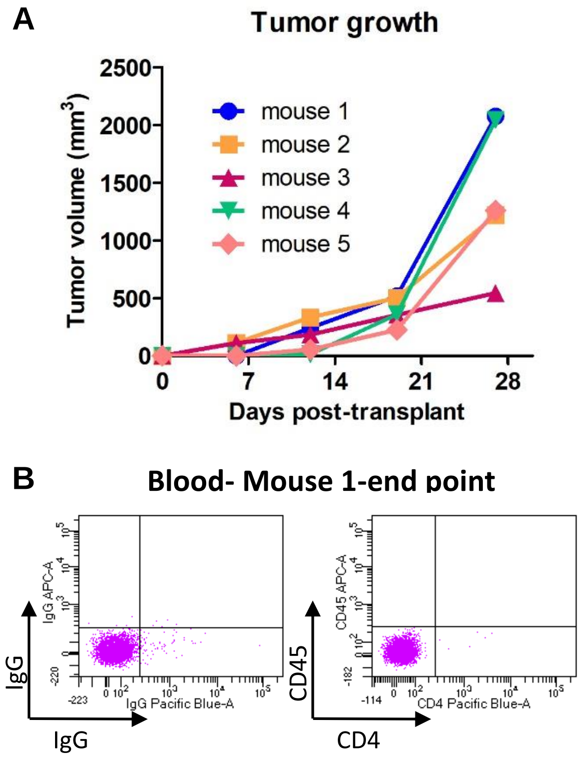

Fig. S5: Percutaneous Hut78 cell xenograft in immunodeficient NSG mice.
$1x10^6$ HUT78 cells were injected percutaneously in NOD SCID IL2Rγc-/- immunodeficient mice.
(**A**) Tumor volume was measured weekly during 4 weeks after transplantation. (**B**) Mice were sacrificed when at least one mouse had a tumor around $2000mm^3$. The white blood cells were prepared and analyzed for CD4 and CD45 expression by FACS. Dot plot represents the expression of human CD4 and CD45 markers and their respective isotype controls for one representative mouse.

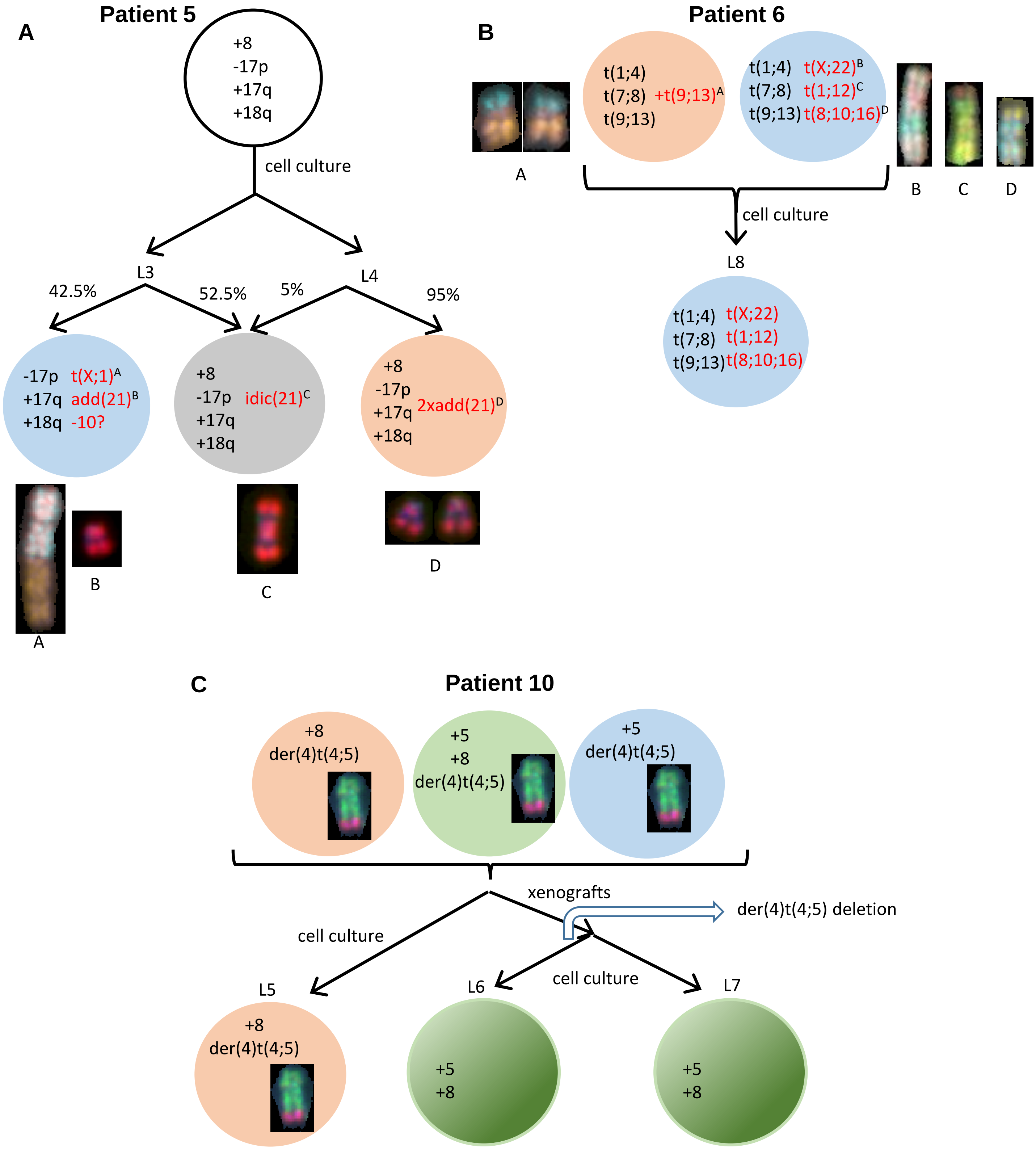

Fig. S6: Clonal heterogeneity and evolution of SCs revealed by PDC and PDX models.
(**A-C**) mFISH and aCGH analyses were performed in patients #5, #6 and #10 derived cells. Schematic representation of the subclonal heterogeneity between original patient samples and after amplification in PDC and/or PDX. Alterations mentioned in black are examples of common clonal alterations. In red are additional alterations distinguishing subclones which are illustrated by mFISH pictures. Circles with different colors distinguish the clones before and after amplification. The blue arrow indicates a loss of this derivative chromosome.

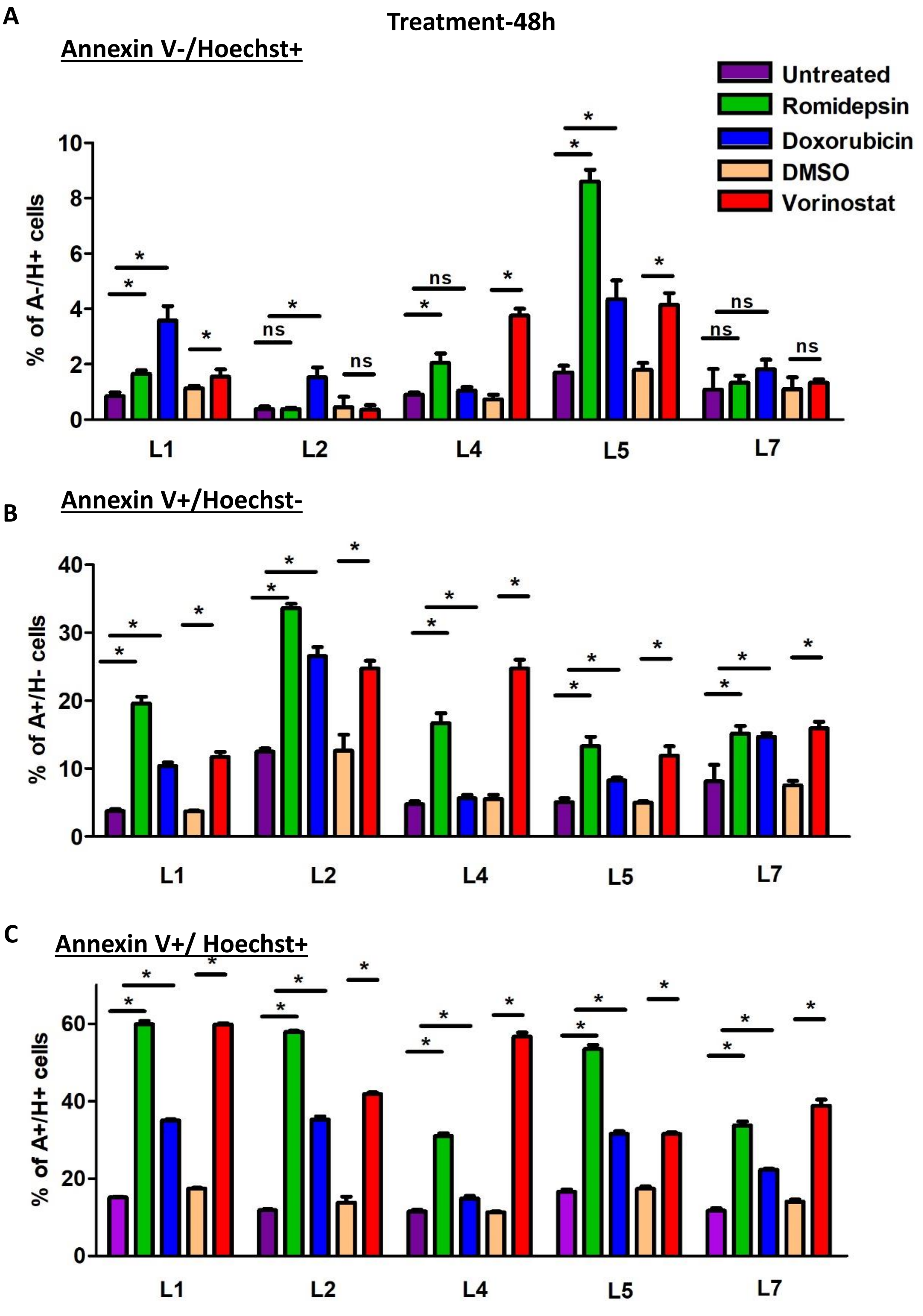

Fig. S7: Therapeutic response to HDAC inhibitors and doxorubicin on SC lines survival. $1x10^6$ cells/well were plated and treated for 48 hours with romidepsin (10nM), doxorubicin (20nM) and vorinostat (3µM). Untreated and DMSO conditions were added as controls. The cell death was evaluated by FACS based on Annexin V and Hoechst stainings. Percentage of (**A**) Annexin V-/Hoechst+ (A-/H+), (**B**) Annexin V+/Hoechst- (A+/H-) and (**C**) Annexin V+/ Hoechst+ (A+/H+) cells in each conditions for 5 SC lines were quantified (mean±SD). Data were analyzed using Mann and Whitney test and were considered statistically significant at $^*P < 0.05$.
