# Supplementary material for: Xenograft and cell culture models of Sézary syndrome reveal cell of origin diversity and subclonal heterogeneity
Source: Leukemia. 2020 Oct 26;35(6):1696–709. doi: 10.1038/s41375-020-01068-2 (PMC8179845; doi:10.1038/s41375-020-01068-2)
Supplement: Supplementary file 3 — Supplementary materials and methods [file 41375_2020_1068_MOESM3_ESM.docx]

**Supplementary Materials and Methods**

**Mice**

For mice engrafted by intrafemoral route, BM sampling each month after anesthesia (isoflurane 2% at 0.2 liter/min) assessed the monitoring of lymphoma engraftment. FACS detected human SC as described in Materials and Methods. Sacrifice was performed at a 50% engraftment threshold of SC cells in BM aspirates or when mice showed clinical signs of illness. For percutaneous engraftment, measurement of the cutaneous nodule was performed weekly until a 2000 mm^3^ volume where mice were sacrificed. After dissection and cell suspension establishment, the percentage of SC was determined in different tissues by FACS. Immunohistochemistry was performed after fixation and paraffin embedding as described below. All procedures were performed in compliance with French Ministry of Agriculture regulations (animal facility registration number: A33063916) for animal experimentation and in accordance with the local ethical committee (Reference number 2016051011358065). The estimation of the sample size for animal experiments is based on previous publications [1,2]. For such animal experiments, no randomization and blinding were necessary.

**Cell line**

Hut 78 cell line (ATCC-TIB-161) were purchased from ATCC and culture as provided by the manufacturer. Hut 78 cell line was routinely test for mycoplasma contamination.

**DNA extraction**

DNA was extracted from patient samples using the QIAmp DNA mini kit (Qiagen, Hilden, Germany) according to manufacturer instructions.

**Antibodies**

Clonal TCRvβ was determined using IOTest® Beta Mark TCRVβ Repertoire Kit according manufacturer recommendations (Beckman Coulter, Villepinte, France). Cells were stained with fluorescein (FITC), phycoerythrin (PE), PE-cyanin 7 (PE-Cy7), allophycocyanin (APC), APC-cyanin 7 (APC-Cy7), Peridinin Chlorophyll Protein Complex-cyanin 5.5 (PercP-Cy5.5) and Brilliant Violet 421 (BV421) conjugated mouse anti-human monoclonal antibodies specific for sCD3, CD4, CD8, CD45RO, CD45, CCR7 (BD Biosciences) and TCRVβ2 or 22 or 13.6 or 7.1 (Beckman Coulter, Villepinte, France). A control staining was performed with their respective isotype control antibodies.

**Immunohistochemistry**

Human leukocyte antigen (HLA-ABC) IHC were performed on 3 μm thick sections (1:100 of Antibody 70328; Abcam, Paris, France) of formalin-fixed, paraffin-embedded tissues with Mayer's hematoxylin counterstaining. Hematoxylin-Eosin-Saffron (HES) stained sections were analyzed in parallel. Images were obtained using a NIKON Eclipse C1 microscope coupled with a camera NIKON DS-FI2 and NIS BR imaging software version 4.0 (Nikon, Champigny sur Marne, France).

**Multicolor fluorescence in situ hybridization (mFISH) karyotyping**

mFISH experiments were conducted using 24XCyte kit (MetaSystems, Altlussheim, Germany). Metaphase were acquired thanks to the Metafer scanning system and images analysed by the Isis software to establish karyotype (MetaSystems).

Briefly, metaphase spreads were prepared one week before hybridization and each slide checked for metaphase concentration. Slides were rehydrated through decreasing ethanol series (100%, 70%, 50%, 30%) and 0.1xSSC, then treated with 2xSSC at 70°C for 30 minutes and let cooled down for next 20 minutes. Finally, slides were transferred to 0.1xSSC, denatured in 0.07M NaOH, passed in 0.1xSSC and 2xSSC at 4°C and dehydrated through increasing ethanol series. Each step was performed at room temperature for 1 minute when not specified. Slides were air dried before applying probe. Probe mixture was denatured by incubating at 75°C for 5 minutes, then put on ice briefly and incubated at 37°C for 30 minutes. Four microlitres of prehybridized probe were applied onto the denatured chromosome preparation and overlaid with 14mm of diameter coverslip, sealed with rubber cement and let hybridized during three days at 37°C in humidified chamber (ThermoBrite, Abbott). Post-hybridization washes were realised in 0.4xSSC at 72°C for 2 minutes, then in 2xSSC / 0.05% Tween-20 for 3 minutes and in PBS for next 2 minutes at RT. Finally, slides were left air dried and mounted with Vectashield (Vector Laboratories) containing 4′,6-Diamidino-2-phenylindole dihydro-chloride (DAPI) in order to counterstain metaphase spreads. Metaphase images were acquired by scanning system Metafer (MetaSystems) on a fully motorized Axio Imager Z2 microscope (Zeiss) equipped with motorized eight-slide scanning stage (Märzhäuser Wetzlar) and high resolution monochrome camera CoolCube 1m (MetaSystems). Slide scanning and automated mFISH acquisition were performed using 63x immersion oil objective and appropriate individual excitation and emission filter sets for six individual fluorochromes (DAPI, Diethyl Aminomethyl Coumarin, DEAC, Fluorescein-Isothiocyanate, FITC,SpectrumOrange, TexasRed, Cyanine5).

A minimum of 10 abnormal karyotypes was established per sample and up to 30 karyotypes analyzed when two or more cytogenetic subclones were present. The presence of an abnormal karyotype at in at least three metaphases defined a subclone.

**Oligonucleotide array-based CGH**

DNA was extracted from patient samples or SC lines and analyzed by high-density array comparative genomic hybridization (CGH) technologies using the 4 x 180K Microarray SurePrint G3 Catalog (Agilent Technologies, Les Ulis, France). The cytogenetic profiles were obtained using the Genomic Workbench software (Agilent Technologies).

**Lymphopanel analysis-data processing**

The panel detailed in Table S5 was designed with Ion Ampliseq technology (ThermoFisher Scientific, Life Technologies, Les Ulis France) and covers 93,13 kbases. Libraries were amplified by emulsion PCR and enriched using automatic system IonChef (ThermoFisher Scientific). Ion sphere particles were then sequenced with the IonS5 (ThermoFisher Scientific) on 530 Chips (ThermoFisher Scientific). The median overall sequencing depth was 3639x [3070x-4920x]. Torrent Suite™ version 5.6 software (ThermoFisher Scientific) was used to perform data processing, alignment to human hg19 reference genome and mutation calling. The Variant Caller detected point mutations with a variant frequency ≥2% for Single Nucleotide Variation (SNV) and ≥5% for short insertion/deletion (INDEL). VCF files generated by Variant Caller were analyzed by ANNOVAR (Wang et al., 2015). BAM sequence were also checked using Alamut Software (Interactive Biosoftware, Rouen, France) if necessary.

**References**

1. Poglio S, Cahu X, Uzan B, Besnard-Guérin C, Lapillonne H, Leblanc T, et al. Rapid childhood T-ALL growth in xenograft models correlates with mature phenotype and NF-κB pathway activation but not with poor prognosis. Leukemia. 2015 Apr;29(4):977–80.

2. Poglio S, Lewandowski D, Calvo J, Caye A, Gros A, Laharanne E, et al. Speed of leukemia development and genetic diversity in xenograft models of T cell acute lymphoblastic leukemia. Oncotarget. 2016 Jul 5;7(27):41599–611.
